# In the Search of Glycoside-Based Molecules as Antidiabetic Agents

**DOI:** 10.1007/s41061-019-0243-6

**Published:** 2019-06-05

**Authors:** Aleksandra Pałasz, Dariusz Cież, Bartosz Trzewik, Katarzyna Miszczak, Grzegorz Tynor, Bartłomiej Bazan

**Affiliations:** Department of Organic Chemistry, Faculty of Chemistry, Jagiellonian University, Gronostajowa 2, 30-387 Kraków, Poland

**Keywords:** *O*-Glycosides, *N*-Glycosides, *C*-Glycosides, Diabetes type 2, Glycogen phosphorylase inhibitor, Sodium-dependent glucose cotransporter inhibitor

## Abstract

This review is an effort to summarize recent developments in synthesis of *O*-glycosides and *N*-, *C*-glycosyl molecules with promising antidiabetic potential. Articles published after 2000 are included. First, the *O*-glycosides used in the treatment of diabetes are presented, followed by the *N*-glycosides and finally the *C*-glycosides constituting the largest group of antidiabetic drugs are described. Within each group of glycosides, we presented how the structure of compounds representing potential drugs changes and when discussing chemical compounds of a similar structure, achievements are presented in the chronological order. *C*-Glycosyl compounds mimicking *O*-glycosides structure, exhibit the best features in terms of pharmacodynamics and pharmacokinetics. Therefore, the largest part of the article is concerned with the description of the synthesis and biological studies of various *C*-glycosides. Also *N*-glycosides such as *N*-(β-d-glucopyranosyl)-amides, *N*-(β-d-glucopyranosyl)-ureas, and 1,2,3-triazolyl derivatives belong to the most potent classes of antidiabetic agents. In order to indicate which of the compounds presented in the given sections have the best inhibitory properties, a list of the best inhibitors is presented at the end of each section. In summary, the best inhibitors were selected from each of the summarizing figures and the results of the ranking were placed. In this way, the reader can learn about the structure of the compounds having the best antidiabetic activity. The compounds, whose synthesis was described in the article but did not appear on the figures presenting the structures of the most active inhibitors, did not show proper activity as inhibitors. Thus, the article also presents studies that have not yielded the desired results and show directions of research that should not be followed. In order to show the directions of the latest research, articles from 2018 to 2019 are described in a separate Sect. 5. In Sect. 6, biological mechanisms of action of the glycosides and patents of marketed drugs are described.

## Introduction

Diabetes mellitus is a disease closely associated with the metabolic syndrome and in developed countries it is a major public health problem [1–3]. There are three main types of diabetes mellitus: type 1 (insulin-dependent), type 2 (insulin resistance), and gestational diabetes. Type 2 diabetes mellitus (T2DM) accounts for 90–95% of the diabetic cases. In T2DM, insulin resistance is the major problem. Chronic hyperglycemia is associated with long-term damage, dysfunction and failure of various organs such as eyes, kidneys, nerves, heart and blood vessels. While type 1 diabetics can be treated by the administration of exogenous insulin, for type 2 patients generally diet, exercise, and oral hypoglycemic agents are prescribed. A large number of oral antidiabetic drugs aimed to eliminate three major metabolic disorders leading to hyperglycemia-dysfunction of β-cells, peripheral insulin resistance, excessive hepatic glucose production [4, 5]. Current pharmacological treatments are symptomatic and aim at maintaining the blood glucose levels close to the fasting normoglycemic range of 3.5–6 mM/l. This can be achieved by an array of small molecule drugs (e.g., biguanides, sulfonylureas, thiazolidinediones, glycosidase inhibitors) and ultimately by administration of insulin.

**Fig. 1:**
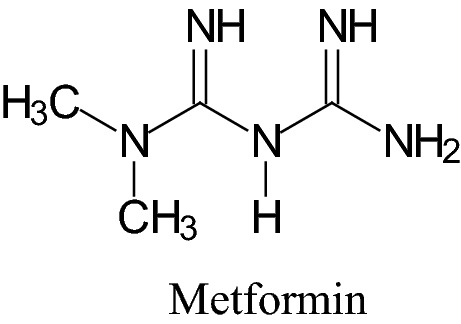
Metformin—the most widely prescribed antidiabetic drug

Metformin is a biguanide, which is now the most widely prescribed antidiabetic drug (Fig. 1). Metformin is the first-line medication for the treatment of type 2 diabetes particularly in people who are overweight and is believed to be the most widely used medication for diabetes, which is taken by mouth. However, for 30–40% of T2DM patients, combination therapy is frequently applied as pharmacological treatments.

Glycogen is a polymer of α-1,4- and α-1,6-linked glucose units that provides a readily available source of energy in living organisms. Glycogen synthase (GS) and glycogen phosphorylase (GP) are the two enzymes that control the synthesis and degradation of this polysaccharide. A key role in glycogen metabolism plays GP [6, 7]. With the rapid increase of type 2 diabetic patients recently, it is becoming an interesting field to discover GP inhibitors for potential antidiabetic drugs. As GP is a typical allosteric protein with several key inhibitor-binding sites including the inhibitor, the catalytic, the allosteric, and the new allosteric sites, the research works were mainly focused on compounds that can bind these sites and show selective inhibitory effect [6–10]. So, GP transfers a glucose unit from the non-reducing end of the storage polysaccharide glycogen to an inorganic phosphate. Three isoforms of GP exist in the brain, muscle, and liver tissue. The liver is capable of storing glucose as glycogen and producing and releasing glucose to the bloodstream [6, 7]. GP is an allosteric enzyme, which exists in two interconvertible forms GPa (phosphorylated, active, high substrate affinity) and GPb (unphosphorylated, inactive, low substrate affinity). Design of GP inhibitors is a target for a better control of hyperglycemia. The inhibitors targeting the seven binding sites of GP show a large molecular diversity. Among them, various glucose derivatives bind mostly to the catalytic site of the enzyme. *N*-Acyl-β-d-glucopyranosylamines, *N*-acyl-*N*′-β-d-glucopyranosyl ureas, glucopyranosylidene-spiro-heterocycles, as well as *N*- and *C*-glucosylated heterocycles belong to the most potent classes of this inhibitor family [6, 7]. In 2001, So and Karplus designed a number of potential GP inhibitors with a variety of computational approaches [11]. 2D and 3D similarity-based QSAR models were used to identify novel molecules that may bind to the glucose-binding site. The designed ligands were evaluated by a multiple screening method [12]. In this way, a total of 301 candidate ligands for GP have been designed using an array of computational approaches.

Several kinds of mimics of *O*-glycosides, first of all *S*-, *N*-, and *C*-glycosyl derivatives, may display similar biological activities; however, due to their significantly distinct chemical properties, such molecules can be valuable tools in deciphering the biological roles of natural sugars, and may also serve as leads for new drugs. Among glycomimetics, *C*-glycosides have attracted much attention due to the existence of a number of naturally occurring representatives. Comparing to *O*-glycosides, the *C*-glycosides are structurally more stable against acidic and enzymatic cleavage due to the existence of their C–C glycosidic bond. Bristol-Myers Squibb [13] and Kotobuki [14] disclosed *C*-aryl glucosides in 2001, which appear to have potent inhibition and good stability in vivo.

**Fig. 2:**
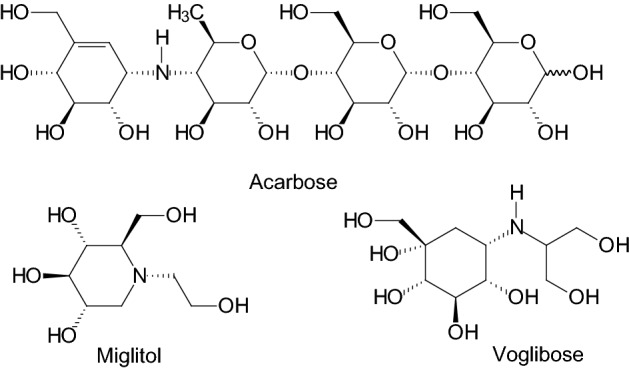
Carbohydrate derivatives and glycomimetic compounds in therapies of T2DM

Many efforts devoted to develop carbohydrate-based therapeutics aim at finding inhibitors of glycoprocessing enzymes and discovering their structure–activity relationships (SAR). In therapies of diabetes, sugar derived or glycomimetic structures, such as acarbose, miglitol, or voglibose, have been applied (Fig. 2) [15].

**Fig. 3:**
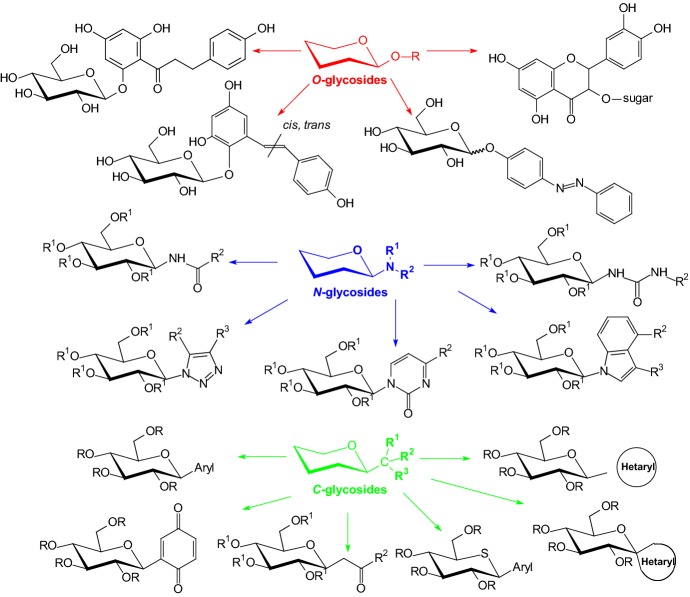
*O*-Glycosides and *N*-, *C*-glycosyl antidiabetic molecules that are discussed in this review

In 2017, Bokor et al. presented a review [16] where they described the syntheses and diverse bioactivities of *C*-glycopyranosyl arenes and heteroarenes. They provided a classification of the preparative routes to these synthetic targets according to methodologies and compound categories. Several of these compounds display antidiabetic properties due to enzyme inhibition and are used in the pharmacological treatment of type 2 diabetes. Figure 3 shows the glycoside structures that are discussed in this article. *O*-Glycosides, *N*-glycosides, and *C*-glycosides as antidiabetic drugs have been described in the following sections.

## *O*-Glycosides as Antidiabetic Agents

**Fig. 4:**
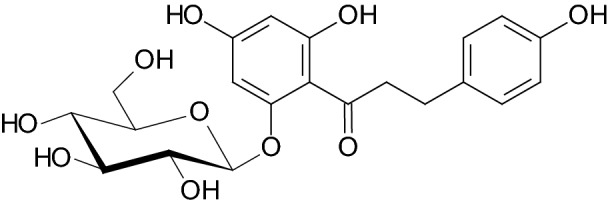
Structure of the natural product phlorizin

It is known that an *O*-glycoside—natural product phlorizin (Fig. 4) can lower plasma glucose levels and improve insulin resistance by increasing renal glucose excretion [17]. However, its sensitivity toward hydrolysis by glucosidases, unselective inhibition of both SGLTs (sodium glucose transporters), and unfavorable effects of its aglycon phloretin on other glucose transporters prevented this compound from use as an antidiabetic drug.

**Fig. 5:**
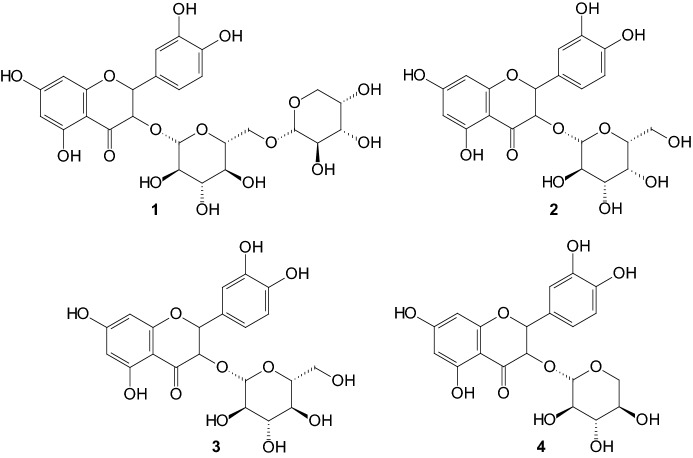
Structures of the flavonol-glycosides: peltatoside **1**, hyperoside **2**, isoquercitrin **3**, guaijaverin **4** [20]

More recently, guava leaves have gained attention in the control of T2DM [18, 19]. In 2013, Eidenberger and coworkers investigated in vitro the effect of extracts from* Psidium guajava* L. leaves containing the flavonol-glycoside components [20]. An ethanolic extract was prepared from dried, powdered leaves of guava and was found to contain seven main flavonol-glycosides, which were isolated by semi-preparative HPLC and tested individually. All isolated flavonol-glycosides were tested for their antidiabetic potential. Peltatoside **1**, hyperoside **2**, isoquercitrin **3**, and guaijaverin **4** (Fig. 5) show an inhibitory effect 5–10 times higher than that obtained for the three other partially characterized flavonol-glycosides. It seems therefore that most of the inhibitory action of the guava extract is due to the four identified flavonol constituents **1**–**4** [20].

**Scheme 1:**
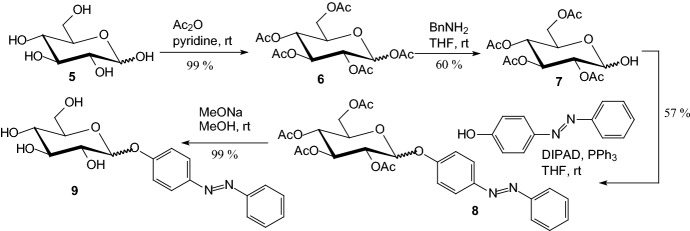
Synthesis of 4-(phenylazo)phenyl-d-glucopyranoside **9** [21]

In 2015, Diaz-Lobo et al. [21] reported on the synthesis and biological evaluation of *O*-glycoside—a selective inhibitor that consists of an azobenzene moiety glycosidically linked to the anomeric carbon of a glucose molecule. The molecule incorporates an azobenzene photoswitch whose conformation can be significantly altered by irradiation with UV light. Synthesis of compound **9** (Scheme 1) started with the quantitative peracetylation of d-glucose **5** with acetic anhydride in pyridine. Next, the anomeric acetyl group of 1,2,3,4,6-*penta*-*O*-acetyl-d-glucopyranoside **6** was selectively cleaved using benzylamine in THF to furnish **7** that was employed for the glycosylation of 4-hydroxyazobenzene by the Mitsunobu reaction. The resulting 4-(phenylazo)phenyl-2,3,4,6-tetra-*O*-acetyl-d-glucopyranoside **8** was deacetylated with MeONa/MeOH to give 4-(phenylazo)phenyl-d-glucopyranoside **9**. The azoglucoside **9** was obtained as a mixture of the α and β anomers. UV light induced *E*→*Z* photoisomerization of the azobenzene glucoside **9** was observed. In the ground state, the more stable (*E*)-isomer of the azobenzene glucoside **9** had a slight inhibitory effect on rat muscle GP (RMGP, IC_50_ = 4.9 mM) and *Escherichia coli* GS (EcGS, IC_50_ = 1.6 mM). After irradiation and subsequent conversion to the (*Z*)-form, the inhibitory potency of the azobenzene *O*-glucoside did not significantly change for RMGP (IC_50_ = 2.4 mM), while its effect on EcGS increased 50-fold (IC_50_ = 32 μM). Although compound **9** was synthesized as a 1:4 mixture of the α- and β-anomers, analysis suggested that the more abundant β-anomer is the one responsible for the observed inhibition. So, Diaz-Lobo et al. showed that the ability to selectively photocontrol the catalytic activity of key enzymes of glycogen metabolism might represent a new approach for the treatment of glycogen metabolism disorders [21].

**Fig. 6:**
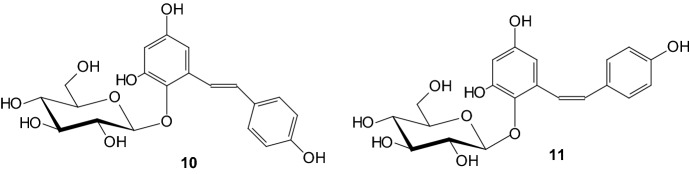
Structures of *trans*-THSG **10** and *cis*-THSG **11** [22]

Functional foods can be used alone or in combination with existing therapies in preventing and treating type 2 diabetes. *Trans*-2,3,5,4′-tetrahydroxystilbene 2-*O*-β-glucopyranoside (*trans*-THSG) **10** (Fig. 6), a dominant bioactive compound from *Polygonum multiflorum* (PM), has attracted increasing research interests due to its strong antioxidant activity. The content of naturally occurring *cis*-THSG (*cis*-2,3,5,4′-tetrahydroxystilbene 2-*O*-β-glucopyranoside) **11** (Fig. 6) is very low in PM root, therefore W. Tang et al. prepared in 2017 *cis*-THSG by mimicking the traditional process of PM [22]. The anti-diabetic effects of *trans*- and *cis*-THSG were evaluated in type 2 diabetes. *Cis*-THSG **11** was found to be more effective than *trans*-THSG **10** in hypoglycemic effect [22].

**Fig. 7:**
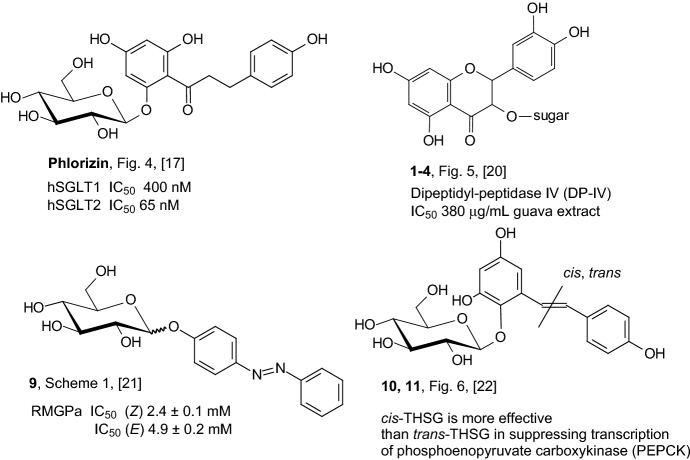
Antidiabetic activity of *O*-glycosides described in Sect. 2

Figure 7 presents information on the antidiabetic activity of *O*-glycosides discussed in Sect. 2. Below the structural formula of each inhibitor, the number of the compound and the scheme number on which it is located and the corresponding reference are provided. The most important information on the action of a given compound as a specific inhibitor is also included. Analyzing the structure of the compounds shown in Fig. 7, it can be seen that the phenyl groups are a structural element that is repeated in each compound. In the case of three compounds, they are phenolic derivatives. An interesting approach to the issue of active inhibitor structure is the idea presented by Diaz-Lobo and coworkers [21], in which they turned their attention to the ability to selectively photocontrol the catalytic activity of key enzymes of glycogen metabolism.

## *N*-Glycosides as Antidiabetic Agents

### *N*-(β-d-Glucopyranosyl) Amides and *N*-(β-d-Glucopyranosyl)-Urea Derivatives

**Fig. 8:**
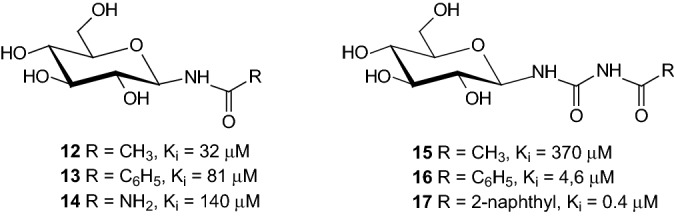
*N*-(β-d-glucopyranosyl) amides **12–14** and *N*-(β-d-glucopyranosyl)-*N*’-acyl urea derivatives **15–17** as inhibitors of GP [23]

Since *O*-glycosides are usually hydrolytically unstable, many carbohydrate analogues such as *N*- or *C*-glycosides have been synthesized as therapeutic agents. Inhibition of GP is one of several intensively investigated approaches to find novel treatments for type 2 diabetes mellitus. Some *N*-glycosides such as *N*-(β-d-glucopyranosyl) amides **12**–**14** (Fig. 8) were examined as inhibitors of GP [23]. *N*-(β-d-glucopyranosyl)-*N*’-acyl urea derivatives **15** and **16** are also inhibitors of GP. Compound **17** represents the most efficient glucose analogue inhibitor [24].

**Scheme 2:**
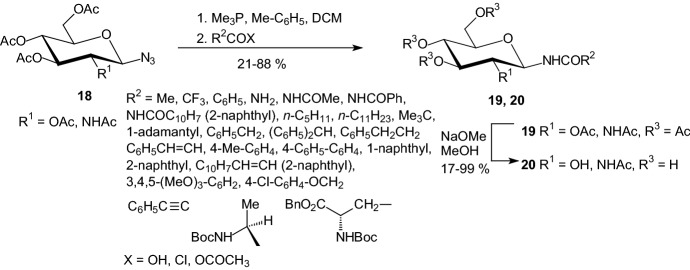
Synthesis of *N*-(β-d-glucopyranosyl)- and *N*-(2-acetamido-2-deoxy-β-d-glucopyranosyl) amides **20** [25]

In 2004, Gyorgydeak et al. transformed 2,3,4,6-tetra-*O*-acetyl-β-d-glucopyranosyl- and 2-acetamido-3,4,6-tri-*O*-acetyl-2-deoxy-β-d-glucopyranosyl azides **18** into the corresponding per-*O*-acetylated *N*-(β-d-glycopyranosyl) amides **19** by Staudinger protocol (Scheme 2) [25]. Removal of the protecting groups carried out by Zemplén deacetylation furnished compounds **20**. Compounds **19** and **20** were tested against rabbit muscle glycogen phosphorylase. The best inhibitor of this series was *N*-(β-d-glucopyranosyl) 3-(2-naphthyl)-propenoic amide (*K*_i_ = 3.5 μM). It was shown that the acyl urea moiety is essential for the strong inhibition. A properly positioned and large enough hydrophobic group attached to the amide moiety makes the inhibition one order of magnitude stronger than that of the best amide inhibitor known earlier [*N*-(β-d-glucopyranosyl) acetamide **12**, Fig. 8]. However, *N*-(β-d-glucopyranosyl) 3-(2-naphthyl)-propenoic amide is still much less efficient than the best-known inhibitor urea derivative **17** (*K*_i_ = 0.4 μM) [25].

**Scheme 3:**
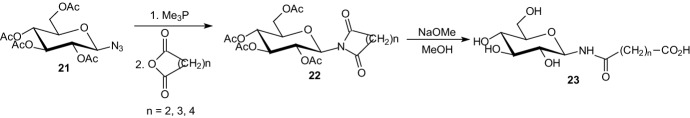
Synthesis of *N*-(β-d-glucopyranosyl) monoamides **23** of various dicarboxylic acids [26]

In 2006, Czifrak et al. used extension of the modified Staudinger methodology to the synthesis of *N*-(β-d-glucopyranosyl) monoamides of various dicarboxylic acids [26]. Such compounds offer the possibility to place a strongly polar group (COOH) at different distances from the sugar moiety while the ability to form the important H-bond from the amide can be maintained. *O*-Peracetylated *N*-(β-d-glucopyranosyl)imino trimethylphosphorane obtained in situ from 2,3,4,6-tetra-*O*-acetyl-β-d-glucopyranosyl azide **21** and PMe_3_ (Scheme 3) was reacted with saturated and unsaturated aliphatic and aromatic dicarboxylic acids, or their anhydrides, or monoesters to give the corresponding *N*-(β-d-glucopyranosyl) monoamides of dicarboxylic acids or derivatives (e.g., derivative **23,** Scheme 3). The acetyl protecting groups were removed according to the Zemplén protocol to give a series of compounds, which were moderate inhibitors against rabbit muscle glycogen phosphorylase *b*. The best inhibitor was 3-(*N*-β-d-glucopyranosyl-carbamoyl) propanoic acid **23** (*n* = 2) with *K*_i_ = 20 μM [26].

**Scheme 4:**
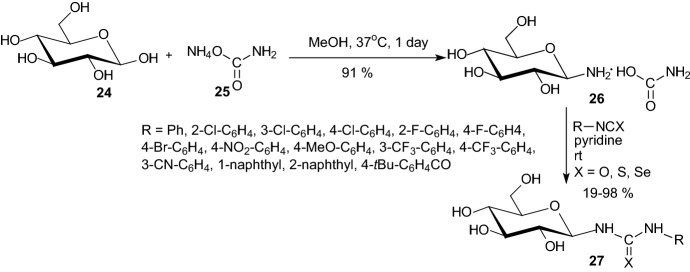
Synthesis of GP inhibitors *N*-(β-d-glucopyranosyl)-*N*’-substituted ureas **27** with using of glucopyranosylammonium carbamate **26** [27]

In 2008, Somsak et al. showed that the synthesis of the highly efficient glycogen phosphorylase inhibitors *N*-(β-d-glucopyranosyl)-*N*′-substituted ureas has been significantly improved by using of glucopyranosylammonium carbamate [27]. This compound allowed the preparation of *N*-(β-D-glucopyranosyl)-*N*′-substituted ureas, -thioureas and selenourea **27** in two steps from d-glucose **24** (Scheme 4).

**Scheme 5:**
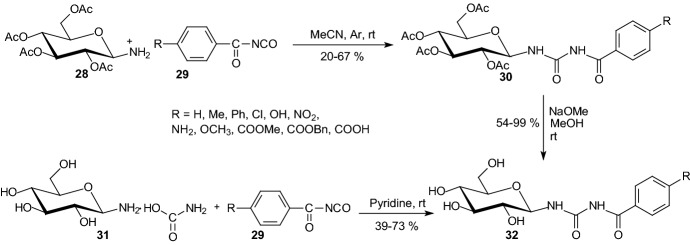
Synthesis of *N*-(4-substituted-benzoyl)-*N*′-(β-d-glucopyranosyl) urea derivatives **32** [28]

In 2012, Nagy et al. synthesized *N*-(4-substituted-benzoyl)-*N*′-(β-d-glucopyranosyl) urea derivatives **32** by addition of *O*-peracetylated β-d-glucopyranosylamine **28** to acyl-isocyanates **29** and subsequent deprotection (Scheme 5) [28]. Some compounds **32** were obtained by reactions of β-d-glucopyranosylammonium carbamate **31** with acyl-isocyanates **29**. Most of the new compounds **32** were low micromolar inhibitors of rabbit muscle glycogen phosphorylase *b*. There was no significant improvement of the inhibitory efficiency for *N*-(4-substituted-benzoyl)-urea **32** in comparison to *N*-benzoyl-urea. These results indicated the lack of a specific and crucial interaction from four position in phenyl ring within the catalytic site. The best inhibitors were compounds **32** with substituents R=4-CH_3_–C_6_H_4_ (*K*_i_ = 2.3 μM) and R=4-NO_2_–C_6_H_4_ (*K*_i_ = 3.3 μM) [28].

**Scheme 6:**
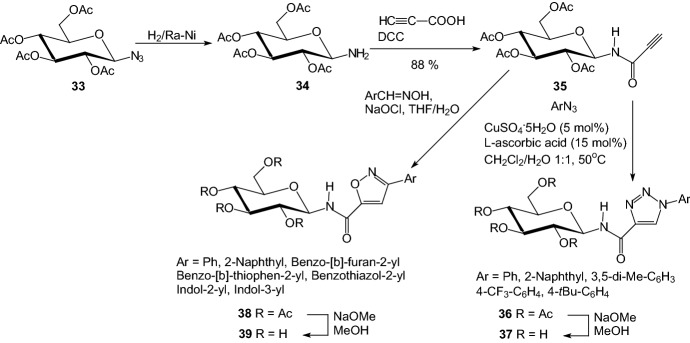
Synthesis of *N*-(β-d-glucopyranosyl)-1-substituted-1,2,3-triazole-4-carboxamides **37** and *N*-(β-d-glucopyranosyl)-3-substituted-isoxazole-5-carboxamides **39** [29]

In 2012, Konya et al. synthesized new glucose derivatives for the inhibition of GP [29]. They have reported on the synthesis and enzymatic evaluation of a series *O*-peracetylated *N*-(*β*-d-glucopyranosyl)-carboxamides with isoxazole or 1,2,3-triazole rings. In a DCC-mediated coupling 2,3,4,6-tetra-*O*-acetyl-*β*-D-glucopyranosylamine **34** and propiolic acid gave *N*-propynoyl-2,3,4,6-tetra-O-acetyl-*β*-d-glucopyranosylamine **35**, which was transformed by 1,3-dipolar cycloadditions with aromatic azides and nitrile-oxides to the corresponding *O*-peracetylated *N*-(β-d-glucopyranosyl)-1-substituted-1,2,3-triazole-4-carboxamides **36** and *N*-(β-d-glucopyranosyl)-3-substitutedisoxazole-5-carboxamides **38**, respectively (Scheme 6). These compounds were *O*-deacetylated by Zemplén protocol to compounds **37** and **39**, which were tested as inhibitors of rabbit muscle glycogen phosphorylase *b*. Deacylated compounds **37** and **39** inhibited rabbit muscle glycogen phosphorylase b in the low micromolar range. The best inhibitors of the two series were *N*-(*β*-d-glucopyranosyl)-1-(3,5-dimethyl-phenyl)-1,2,3-triazole-4-carboxamide **37** (Ar=3,5-di-Me-C_6_H_3_, *K*_i_ = 34 μM) and *N*-(β-d-glucopyranosyl)-3-(indol-2-yl)-isoxazole-5-carboxamide **39** (Ar=indol-2-yl, *K*_i_ = 164 μM).

**Scheme 7:**
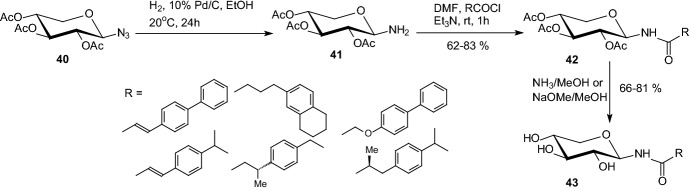
Synthesis of *N*-acyl-β-d-glucopyranosylamines **43** [30]

In 2014, Parmenopoulou and coworkers reported the in silico screening in the Zinc database of 1888 *N*-acyl-β-d-glucopyranosylamines as potential GP inhibitors [30]. Six selected candidates from the screening were then synthesized and their inhibitory potency was assessed both in vitro and ex vivo. The direct acylation of 2,3,4,6-tetra-*O*-acetyl-β-d-glucopyranosylamine **41**, easily prepared from the per-*O*-acetylated β-d-glucopyranosyl azide **40** upon catalytic hydrogenation, with a diverse set of commercially available acyl chlorides RCOCl, furnished the protected *N*-acyl-β-d-glucopyranosylamines **42**. Removal of the acetyl groups of the derivatives **42**, performed either by saturated methanolic ammonia or by the Zemplén method yielded analogues **43** (Scheme 7). Their inhibition constants’ values *K*_i_ in vitro ranged from 5 to 377 μM while two of them were effective at causing inactivation of GP in rat hepatocytes at low μM concentrations [30].

**Fig. 9:**
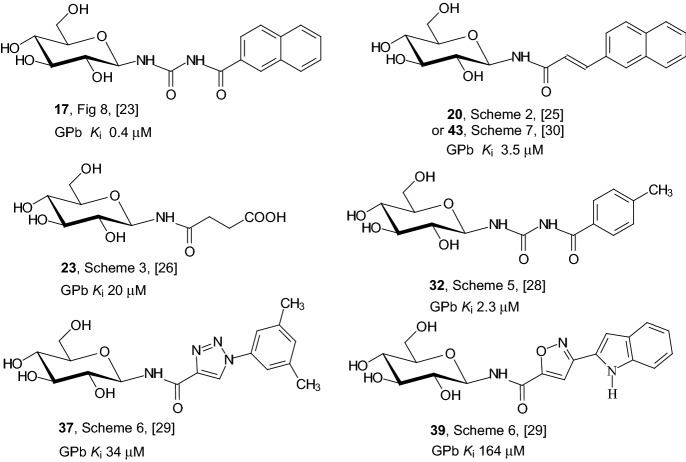
Values of inhibitory constants *K*_i_ of the best GPb inhibitors from the *N*-glycosides described in Sect. 3.1

Figure 9 presents the best GPb inhibitors from the *N*-(β-copper-catalyzed azide–alkyne cycloaddition-glucopyranosyl) amides and *N*-(β-d-glucopyranosyl)-urea derivatives described in Sect. 3.1. The structural formula of each inhibitor, the number of the compound, the scheme number and the corresponding reference are provided. In the figure, the inhibitors are arranged in order from the strongest characterized by the lowest inhibitory constant *K*_i_ value to the weaker one with the highest *K*_i_ value. It can be seen that all inhibitors accumulated in Fig. 8 are derivatives of glucose and compound **17** represents the most efficient glucose analogue inhibitor.

### 1,2,3-Triazolyl *N*-Glycosides

**Scheme 8:**
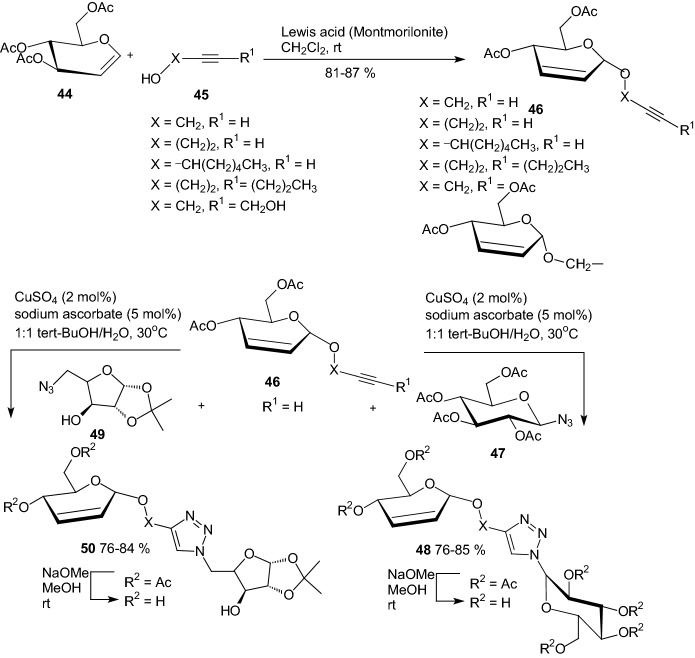
Synthesis of 1,2,3-1*H*-triazolyl glycohybrids **48** and **50** by 1,3-dipolar cycloaddition of glycosyl azides **47** and **49** to 2,3-unsaturated alkynyl glycosides **46** [31]

The medicinal importance of triazoles is due to their bioisosterism with peptide bonds as they can actively participate in hydrogen bonding, and due to their strong dipole moments, the triazoles are extremely stable to hydrolysis and oxidative/reductive conditions. In 2011, Anand and coworkers described an efficient synthesis of 1,2,3-1*H*-triazolyl glycohybrids with two sugar units via copper-catalyzed azide-alkyne cycloaddition (CuAAC) [31]. Potential inhibitors were prepared by a 1,3-dipolar cycloaddition of glycosyl azides **47** and **49** to 2,3-unsaturated alkynyl glycosides **46** (Scheme 8). The synthesized glycohybrids were screened for their α-glucosidase, glycogen phosphorylase, and glucose-6-phosphatase inhibitory activities. A few of the glycohybrids showed promising inhibitory activities against these enzymes [31].

**Scheme 9:**
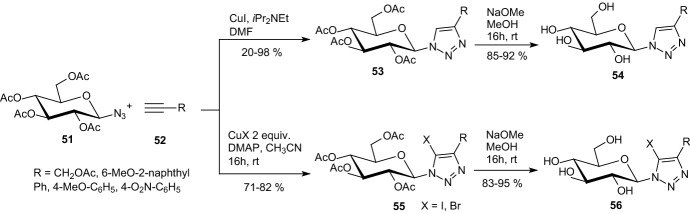
Synthesis of 4-substituted-1-glucosyl-1,2,3-triazoles **54** and **56** by copper-catalyzed azide–alkyne cycloaddition [32]

**Scheme 10:**
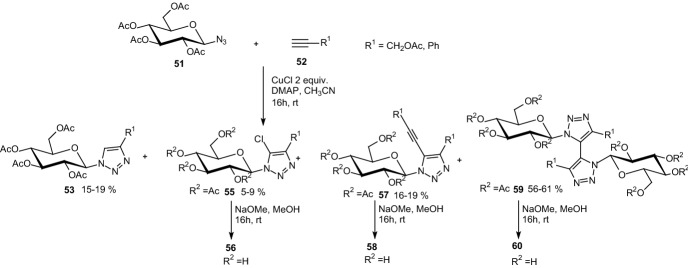
Cycloaddition of azide **51** and acetylenes **52** in the presence of 2 equiv CuCl [32]

In 2014, Goyard et al. examined CuAAC between acetylated β-d-glucosyl azide **51** and alkyl or phenyl acetylenes **52**, which led to the corresponding 4-substituted 1-glucosyl-1,2,3-triazoles **54** (Scheme 9) [32]. 5-Halogeno analogues **56** were prepared in similar conditions but with 2 equiv CuI or CuBr. In reactions with two equiv CuCl and either propargyl acetate or phenyl acetylene, the major products **59** displayed two 5,5′-linked triazole rings resulting from homocoupling of the 1-glucosyl-4-substituted 1,2,3-triazoles (Scheme 10). The cycloaddition of **51** and **52** afforded four different products **53**, **55**, **57**, and **59** with only minor amounts of the expected chlorinated derivatives **55** and with the dimeric products **59** being isolated as the major component. The two 4-phenyl substituted structures of compound **59** were unambiguously identified as atropisomers with *a*R stereochemistry. All *O*-unprotected derivatives (Schemes 9, 10) were tested as inhibitors of GP. The modest inhibition activities measured showed that 4,5-disubstituted 1-glucosyl-1,2,3-triazoles bind weakly to the enzyme. This suggests that such ligands do not fit the catalytic site or any other binding site of the GP [32].

**Scheme 11:**
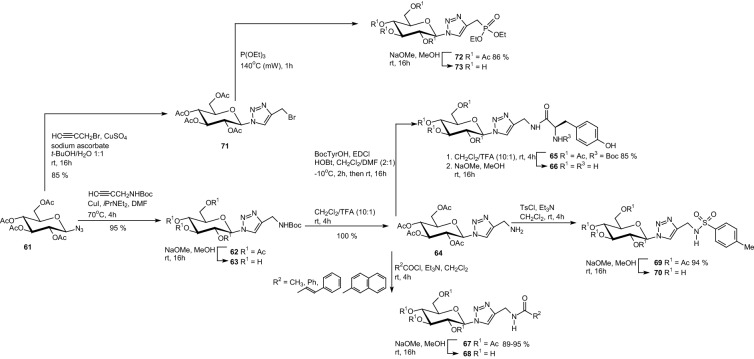
Synthesis of 4-amidomethyl-1-glucosyl-1,2,3-triazoles [33]

In order to gain additional data for structure–activity studies of the inhibition glycogen phosphorylase, in 2015 Goyard et al. prepared a series of eight GP inhibitor candidates from peracetylglucopyranosyl azide **61** by 1,3-dipolar cycloaddition (Scheme 11) [33]. The need for a *N*-Boc-protected propargylamine was identified in the CuAAC with azide **61** under Meldal’s conditions, while Sharpless’ conditions were better adapted to the CuAAC of azide **61** with propargyl bromide. Cycloaddition of Boc-propargylamine with azide **61** afforded the *N*-Boc precursor of a 4-aminomethyl-1-glucosyl-1,2,3-triazole **62**, which gave access to a series of amide and sulfonamide derivatives (Scheme 11). The Boc-protected amine **62** was converted to the free amine **64**, which were functionalized with acyl chlorides R_2_COCl affording the amides **67**. The amine **64** was also converted to the sulfonamide derivative **69** using *p*-toluenesulfonyl chloride TsCl. The sulfonamide **70** was synthesized in order to take advantage of hydrophobic contact in the β-channel of GP and also to have potential additional contacts with the sulfonamide group and the side chain amino acids of the enzyme. Arbuzov reaction of the brominated derivative **71** with triethylphosphite under microwave activation allowed for formation of the acetylated phosphonate **72**, which was converted to phosphonate **73** (Scheme 11). Enzymatic studies revealed poor to moderate inhibitions of deacetylated derivatives toward glycogen GP. The *N*-Boc-protected amine **63** was the best inhibitor (IC_50_ = 620 μM) unexpectedly slightly better than the 2-naphthylamido **68** substituted analogue (IC_50_ = 650 μM) [33].

**Scheme 12:**
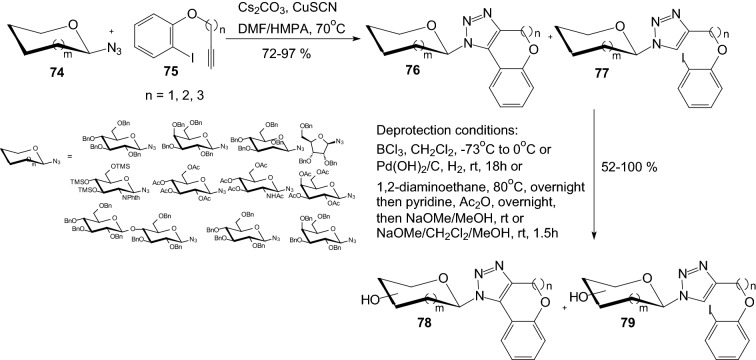
Synthesis of triazole-*N*-glycoside derivatives **78** via CuSCN-catalyzed click reaction and Ullmann-type coupling reaction [34]

SGLT2 (sodium-dependent glucose co-transporter 2) is a glucose transporter that is responsible for 90% of the renal glucose reabsorption. For the treatment of type 2 diabetes, suppression of glucose reabsorption through the inhibition of SGLT2 is a promising therapeutic approach. Therefore in 2015, Bai et al. developed a convenient approach to the synthesis of novel triazole-*N*-glycoside derivatives **78** via CuSCN-catalyzed click reaction and Ullmann-type coupling reaction (Scheme 12) and examined the SGLT2 inhibitory activities of prepared *N*-glycosides [34]. For carbohydrate azides **74**: glucosyl azide, galactosyl azide, ribosyl azide and aminoglucosyl azide, reactions were performed and the triazole-*N*-glycosides **76** were generated with high selectivity, while mannosylazide and lactosyl azide showed moderate selectivity (side product **77**). After the successful construction of [6] ring-fused triazole-*N*-glycosides, the preparation of [6, 7] or [6, 8] ring-fused triazole-*N*-glycosides via this protocol was also tried. Deprotection was carried out in the presence of BCl_3_ or 1,2-diaminoethane and finally the acetyl-containing *N*-glycosides were treated with sodium methoxide to obtain the corresponding target compounds **78** and side product **79** (Scheme 12). The SGLT2 inhibitory activities of *N*-glycosides **78** were evaluated and some compounds showed moderate SGLT2 inhibition activities at 100 nM [34].

### Other *N*-Glycosides

**Scheme 13:**
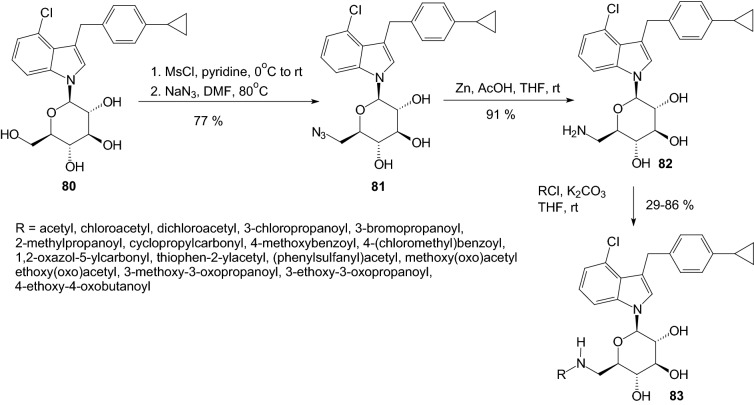
Synthesis of *N*-indolylglucosides **83** [35]

In 2016, Chu et al. investigated the effect of C6-substitution on inhibition of SGLT2 by *N*-indolylglucosides **83** (Scheme 13) [35]. As they investigated in a previous article [36], results suggested that the C6 position of the sugar moiety may play a critical role in the suppression of SGLT2. Therefore, Chu et al. led optimization of *N*-glycosides proceeded via modification at the C6 position only, the aglycone unit 4-chloro-3-(4-cyclopropylbenzyl)-1*H*-indole being fixed. *N*-Indolylglucosides **83** were prepared according to a synthetic method depicted in Scheme 13, using *N*-indolylglucoside **80** as the starting material. Treatment of **80** with methanesulfonyl chloride (MsCl) in pyridine gave the corresponding 6-OMs *N*-glycoside, which was sequentially reacted with sodium azide (NaN_3_) to afford the 6-azido compound **81**. Next, the synthesis of amides **83** was carried out via the amine **82**, generated by the reduction of azido group in **81** with Zn/AcOH in THF. Amine **82** underwent direct amide bond formation with a variety of acyl chlorides to furnish 6-amide derivatives **83** (Scheme 13). After SAR study 6-amide derivatives **83** (R=acetyl and 3-methoxy-3-oxopropanoyl) were identified as potent SGLT2 inhibitors. The data obtained indicated that **83** (R=acetyl and 3-methoxy-3-oxopropanoyl) are mildly to moderately selective for SGLT2 over SGLT1. Both compounds were also evaluated in a urinary glucose excretion test and pharmacokinetic study. Compound **83** (R=acetyl) was found capable of inducing urinary glucose excretion in rats [35].

**Scheme 14:**
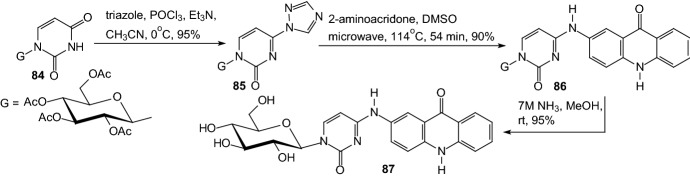
Synthesis of GLAC **87** (N^1^-(*β*-d-glucopyranosyl)-N^4^-[2-acridin-9(10*H*)-onyl]-cytosine) [37]

A key point for the design of efficient drugs is the characterization of the interactions governing its binding to the enzyme. In 2017, Mamais et al. designed and prepared a glucose-based acridone derivative (GLAC), which is a potent inhibitor of GP and it allows probing subtle interactions in catalytic site [37]. The design of a catalytic site inhibitor was based on C-4 modification of β-d-glucopyranosyluracil and introducing of flat aromatic substituent, which can increase the binding affinity in the β-channel. Authors utilized an acridone moiety as a possible chromophore as well as fluorophore. β-d-Glucopyranosyluracil **84** was converted to 4-triazolyl derivative **85** and next substitution of **85** by 2-aminoacridone provided the protected adducts **86** (Scheme 14). Final deprotection furnished desired product GLAC **87**. Authors reveal that the part of the catalytic site of GP behaves as a highly basic environment in which GLAC **87** exists as a bis-anion. Authors reassumed that solvent structure of GLAC and the water-bridged hydrogen bonding interactions formed with the catalytic site residues in the β-channel are responsible for the observed inhibition potency [37].

**Fig. 10:**
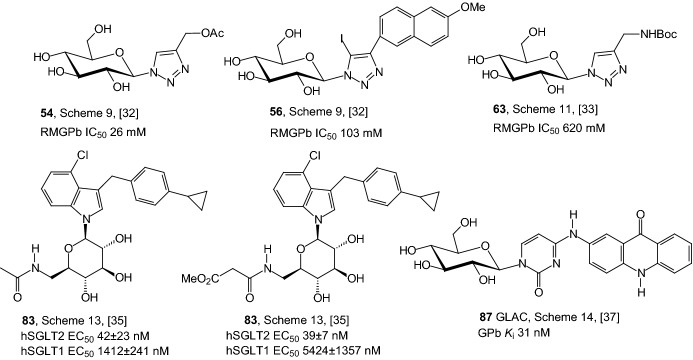
Inhibitory properties of the best GPb and SGLT inhibitors from the *N*-glycosides described in Sects. 3.2 and 3.3

Figure 10 shows inhibitory properties of the best GPb and SGLT inhibitors from the *N*-glycosides: 1,2,3-triazolyl *N*-glycosides, *N*-indolylglycosides, and *N*-uracil glycoside presented in Sects. 3.2 and 3.3. The GLAC compound **87** is one of the best GPb inhibitors described so far (*K*_i_ 31 nM). All compounds are derivatives of glucose. The presence of the cyclopropane ring in the structure of two of the active inhibitors is also noteworthy.

## *C*-Glycosides as Antidiabetic Agents

### Aromatic *C*-Glycosyl Derivatives

**Scheme 15:**
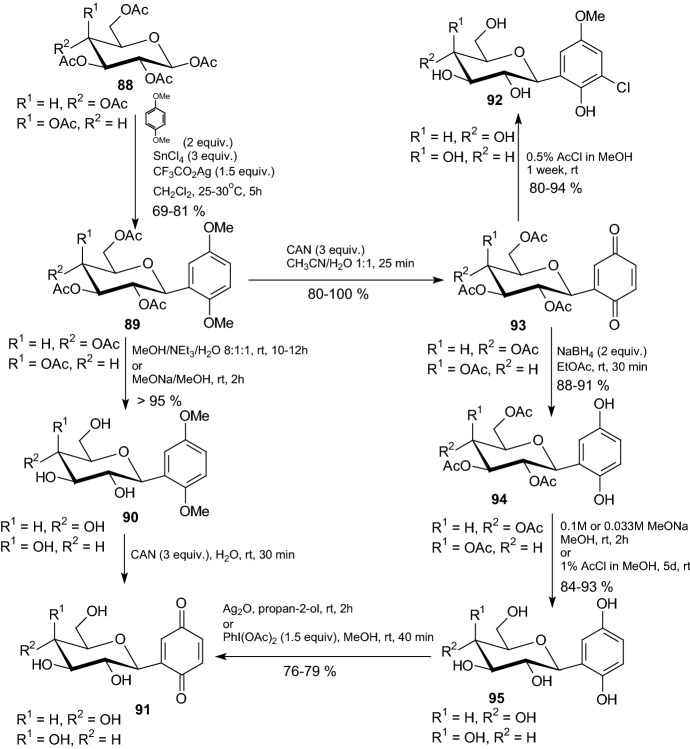
Synthesis of *C*-β-d-glucosylbenzoquinones **91** and *C*-β-d-glucosylhydroquinones **95** [38]

*C*-Glycosides are more metabolically stable than *O*-glycosides and tend to have higher oral bioavailability and plasma exposure without needing to be converted to a prodrug [4]. Among *C*-glycosyl derivatives, *C*-glycosylarenes have attracted much attention. In 2007, Praly et al. underwent kinetic and X-ray crystallographic study of two enzyme complexes of rabbit muscle unphosphorylated glycogen phosphorylase b (GPb) with ligands of the *C*-glucosylbenzo(hydro)quinone type [38]. The synthesis of quinones was accomplished from *C*-β-d-glycopyranosyl-1,4-dimethoxybenzenes **89**, which were prepared by reaction of* penta*-*O*-acetyl-β-d-glycopyranoses **88** and 1,4-dimethoxybenzene (Scheme 15). Next, compounds **89** were converted to the corresponding *C*-glycosylhydro and *C*-glycosylbenzoquinones, with either an acetylated or deprotected sugar moiety. *C*-β-d-Glucosylbenzoquinone **91** (R^1^=H, R^2^=OH) and C-β-d-glucosylhydroquinone **95** (R^1^=H, R^2^=OH) (Scheme 15) were found to be competitive inhibitors of rabbit muscle GPb with *K*_i_ values of 1.3 and 0.9 mM, respectively. In order to elucidate the structural basis of inhibition, the authors determined the crystal structures of **91** and **95** in complex with GPb. The complex structures reveal that the inhibitors can be accommodated at the catalytic site at approximately the same position as α-d-glucose and stabilize the transition state conformation of the 280 s loop by making several favorable contacts to Asp283 and Asn284 of this loop [38].

**Scheme 16:**
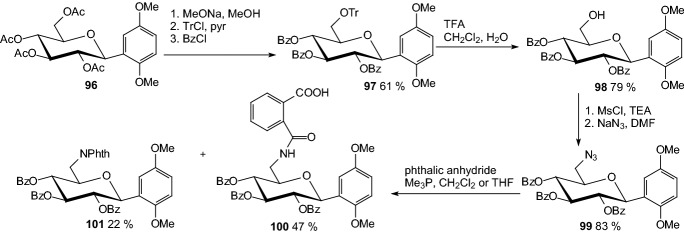
Synthesis 2-carbamoylbenzoic acid derivative of β-*C*-glycosyl compound **100** as sugar-based PTP1B inhibitor [40]

**Scheme 17:**
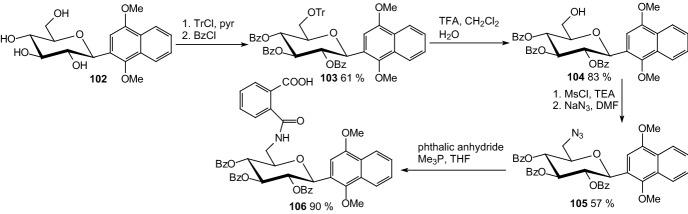
Synthesis 2-carbamoylbenzoic acid derivative of β-*C*-glycosyl compound **106** as sugar-based PTP1B inhibitor [40]

Protein tyrosine phosphatase 1B (PTP1B) has recently been identified as a new drug target for type 2 diabetes [39]. In 2008. Lin et al. synthesized β-*C*-glycosiduronic acid quinones and β-*C*-glycosyl compounds as sugar-based PTP1B inhibitors [40]. To prepare 2-carbamoylbenzoic acid derivatives **100** (Scheme 16) and **106** (Scheme 17), β-*C*-aryl glucosides **96** and **102** were first tritylated at 6-position, followed by protection of secondary hydroxyl function as benzoyl ester. To avoid intramolecular transesterification reaction, detritylation has been realized under acidic condition with TFA to afford **98** and **104**. The 6-hydroxy group was transformed into azide via mesylate. Staudinger protocol was then employed to convert azido sugars to carbamoylbenzoic acid derivatives. Reaction of **99** with phthalic anhydride led to a mixture of the desired compound **100** and *N*-phthalimide derivative **101** (Scheme 16). Treatment of **105** with phthalic anhydride in THF afforded **106** (Scheme 17). Benzoyl protected quinone derivatives as well as aryl β-*C*-glycosyl compounds showed IC_50_ values of 0.77–5.27 μM against PTP1B, with compounds **100** and **106** bearing an acidic function being the most potent [40].

**Scheme 18:**
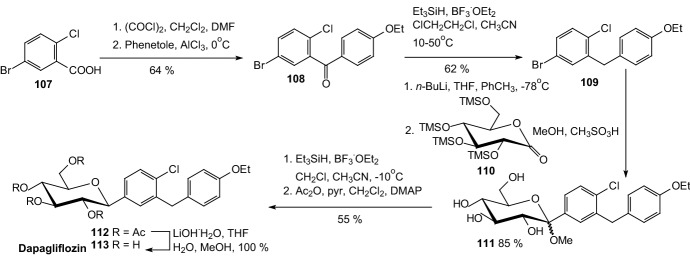
Synthesis of dapagliflozin **113** SGLT2 inhibitor [41]

In 2008, Meng et al. discovered dapagliflozin, selective renal sodium-dependent glucose cotransporter 2 (SGLT2) inhibitor for the treatment of type 2 diabetes [41]. Synthesis of dapagliflozin started from Friedel–Crafts acylation of phenetole with 5-bromo-2-chlorobenzoyl chloride, which was formed from 5-bromo-2-chlorobenzoic acid **107** in reaction with oxalyl chloride (Scheme 18). Reduction of *p*-benzophenone **108** by triethylsilane and BF_3_·OEt_2_ provided aglycon **109**. Lithium halogen exchange, followed by the addition of the nascent lithiated aromatic to **110**, gave a mixture of lactols, which were converted in situ to the desilylated *O*-methyl lactols **111** by treatment with methanesulfonic acid in methanol. Reduction of the anomeric methoxy group of **111** using triethylsilane and BF_3_·OEt_2_, followed by peracetylation, yielded tetraacetate **112**. Hydrolysis of **112** with lithium hydroxide generated **113** (Scheme 18). Authors resumed that dapagliflozin **113** is a potent, selective SGLT2 inhibitor that is not subject to *O*-glucosidase degradation. Compound **113** is a much more potent stimulator of glucosuria in normal rats than other SGLT2 inhibitors. The promising significant reduction of blood glucose levels in diabetic rats prompted further evaluation of **113** in the clinic for the treatment of type 2 diabetes [41].

**Scheme 19:**
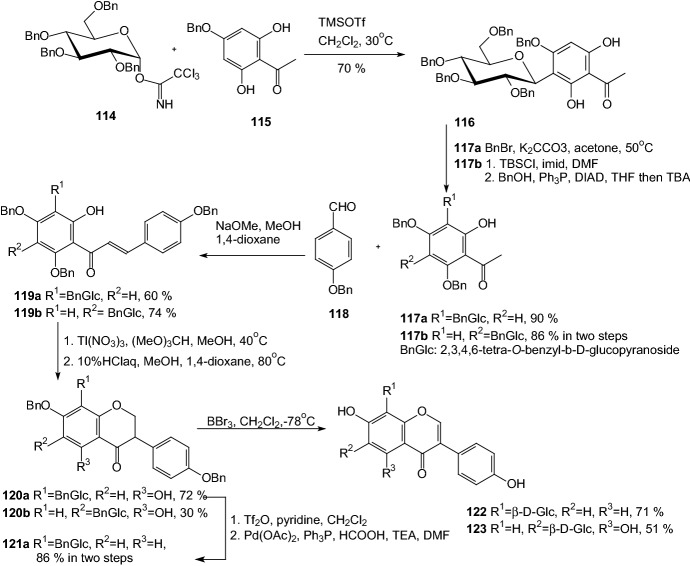
Synthesis of puerarin **122** and its regioisomer **123** [42]

In 2010, Kato and Kawabata synthesized an isoflavone *C*-glucoside puerarin and several derivatives, which were candidate for treatment of diabetes mellitus [42]. Treatment of TMSOTf to the mixture of glucosyl imidate **114** and an acetophenone **115** afforded a *C*-glucoside **116** (Scheme 19). Group 6-OH of compound **116** was selectively protected by a benzyl group. Aldol condensation of **117** with an aldehyde **118** gave chalcones **119**. Compounds **119** were treated with Tl(NO_3_)_3_ and heated in an acidic medium to form isoflavone structure **120**. The benzyl group protecting OH was selectively removed and after trifluoromethanesulfonylation and subsequent reaction with Pd(OAc)_2_ derivative **121a** was prepared. Finally, benzyl groups were removed by treatment with BBr_3_ to give puerarin **122** in 13% overall yield and regioisomer of **122**, genistein 6-*C*-glucoside **123** (Scheme 19). The compound **122** was applied for the structure–activity relationship study. The results of research indicated that the *C*-glucoside part of the compound **122** was not much involved in the activity. The structure responsible for the glucose uptake enhancing activity was the isoflavone moiety. However, the *C*-glucose may involve in physical properties of **122** and raises solubility in water [42].

**Scheme 20:**
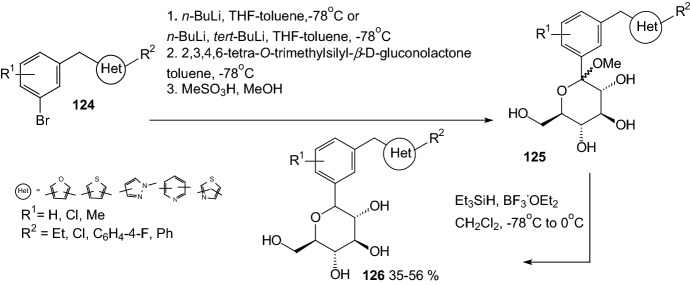
Synthesis of *C*-glucoside derivatives **126** selective SGLT2 inhibitors [43]

In 2010, Nomura et al. discovered that *C*-glucosides bearing heterocyclic ring formed metabolically more stable inhibitors for sodium-dependent glucose cotransporter 2 (SGLT2) than the *O*-glucoside [43]. To synthesize appropriate compounds, aglycones **124** were dissolved in tetrahydrofuran and toluene, and treated with *n*-butyllithium at − 78 °C to generate aryllithium, followed by addition of 2,3,4,6-tetra-*O*-trimethylsilyl-β-d-gluconolactone (Scheme 20). The resulting anomeric mixture of lactols was converted into desilylated methyl ethers **125** by addition of methanesulfonic acid in methanol. *C*-Glucoside derivatives **126** were obtained by stereoselective reduction of **125** using a combination of triethylsilane and boron trifluoride etherate in methylene chloride. Thiophene derivative **126** canagliflozin (R^1^=Me in the* para* position relative to glucose moiety, Het=tiophene with R^2^=C_6_H_4_-4-F in position 2) was a highly potent and selective SGLT2 inhibitor and showed pronounced anti-hyperglycemic effects in high-fat diet-fed mice (IC_50_ = 2.2 nM). Canagliflozin is the first SGLT2 inhibitor to be approved in the USA and is under regulatory review in the EU [44, 45].

**Scheme 21:**
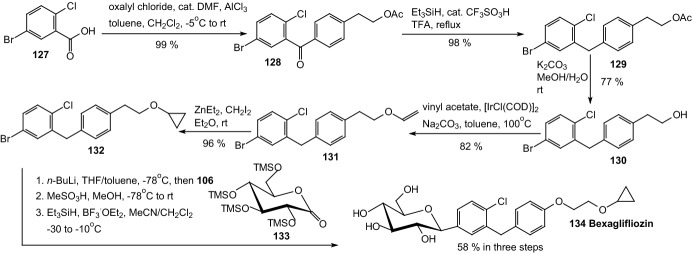
Synthesis of bexagliflozin **134** selective SGLT2 inhibitor [47]

In 2011, Xu and coworkers synthesized a series of *C*-aryl glucosides with various substituents at the 4′-position of the distal aryl ring and evaluated for inhibition of human hSGLT1 and hSGLT2 [46]. Scheme 21 depicts the construction of aglycone **132**, which is a part of bexagliflozin **134** selective SGLT2 inhibitor that reached phase III clinical trials [47]. Friedel–Crafts acylation of benzene with the benzoyl chloride derived from benzoic acid **127** by treatment with oxalyl chloride provided the corresponding benzophenone **128**, which were reduced by triethylsilane in the presence of TFA and catalytic trifluoromethanesulfonic acid to generate bromodiarylmethane **129**. Finally, aglycone **132** was constructed by vinyl ether formation of the alcohol **130** with vinyl acetate in the presence of sodium carbonate and a catalytic amount of [IrCl(COD)]_2_ (Scheme 21). Lithium-bromide exchange of bromodiarylmethane **132** and addition of the resulting aryllithium to 2,3,4,6-tetra-*O*-trimethylsilyl-d-gluconolactone **133** followed by etherification with methanol in the presence of methylsulfonic acid provided desilylated *O*-methyl lactols, which were reduced with triethylsilane and BF_3_.OEt_2_ to give desired *C*-aryl glucoside **134**. The IC_50_ values for bexagliflozin against human SGLT1 and SGLT2 are 5.6 μM and 2 nM, respectively [47].

**Scheme 22:**
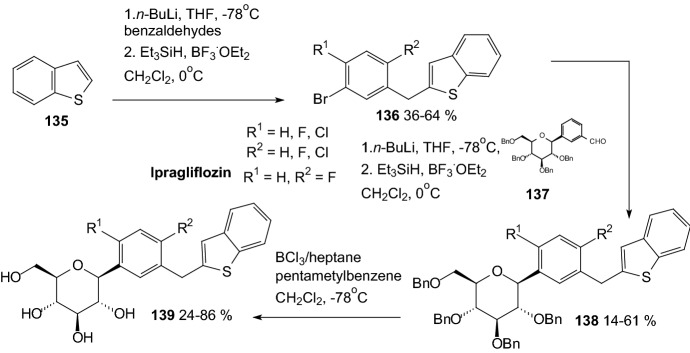
Synthesis of benzothiophene derivatives **139** selective SGLT2 inhibitors [48]

In 2012, Imamura and coworkers discovered a novel benzothiophene derivatives **139**, among them ipragliflozin (R^1^=H, R^2^=F), which are a highly potent and selective SGLT2 inhibitors (Scheme 22) [48]. Lithiation of **135** followed by the addition of aromatic benzaldehydes, yielded alcohols those were reduced with Et_3_SiH and BF_3_.OEt_2_ to give aglycones **136**. Lithium halogen exchange followed by the addition of a lithiated aromatic to compound **137** yielded lactols those were reduced by treatment with Et_3_SiH and BF_3_ etherate to give compounds **138**. Successive removal of the benzyl groups generated compounds **139**. Ipragliflozin (R^1^=H, R^2^=F) was a highly potent and selective human SGLT2 inhibitor (IC_50_ = 7.4 nM).

**Fig. 11:**
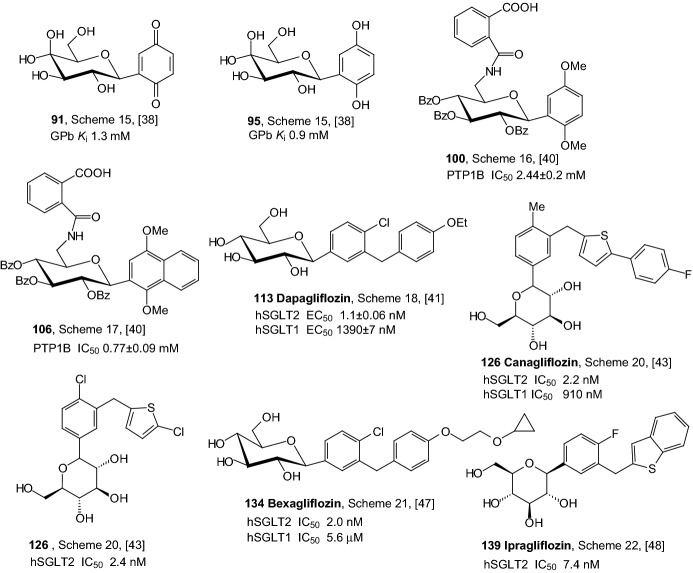
Inhibitory properties of the best inhibitors from the aromatic *C*-glycosyl derivatives described in Sect. 4.1

Figure 11 contains the best inhibitors GPb, PTP1B, and SGLT2 having the aromatic *C*-glycoside structure that are described in Sect. 4.1. The analysis of the structure of the *C*-glycosyl derivatives presented in Fig. 11 shows that the presence of a chlorine or fluorine atom as well as the presence of sulfur-containing heteroaromatic ring guarantees an increased antidiabetic activity of the compound. All inhibitors shown in Fig. 11 are derivatives of glucose. Thus, also taking into account the structures of previously presented the best inhibitors, it can be concluded that this glucose molecule guarantees high inhibitor activity. Referring to the structure of the best inhibitors shown in Fig. 10, it can also be concluded that the cyclopropane ring is a structural element that guarantees high inhibitor activity. The repeating structural element is also the 1,4-dihydroxy or dimethoxyphenyl system.

### Heteroaromatic *C*-Glycosyl Derivatives

**Scheme 23:**
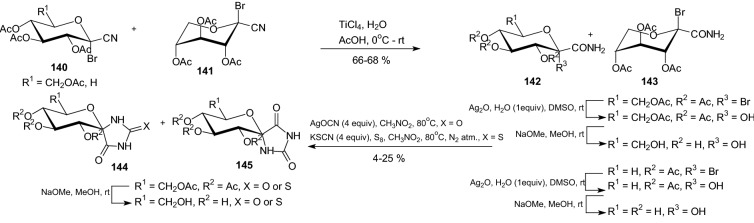
Synthesis of glycopyranosylidenespiro-hydantoins and thiohydantoins **144** and **145** [49]

In 2001, Somsák et al. described highly chemo-, regio-, and stereoselective procedure that allows for the preparation of d-gluco- and d-xylopyranosylidene-spiro-hydantoins and thiohydantoins in six steps from the corresponding free sugar [49]. In the key step of the syntheses *C*-(1-bromo-1-deoxy-β-d-glycopyranosyl)formamides **142** and **143** were reacted with cyanate ion to give spiro-hydantoins **144** and **145** with a retained configuration at the anomeric center as the major products (Scheme 23). Thiocyanate ions gave spiro-thiohydantoins **144** with an inverted anomeric carbon as the only products. The acetylated compounds were deprotected by the Zemplen procedure. Enzyme assays with a and b forms of muscle and liver glycogen phosphorylases showed spiro-hydantoin **144** (R^1^=CH_2_OH, R^2^=H, X=O) and spirothiohydantoin **144** (R^1^=CH_2_OH, R^2^=H, X=S) to be the best and equipotent inhibitors with *K*_i_ values in the low micromolar range. The study of epimeric pairs of d-gluco and d-xylo spiro-hydantoins and *N*-(d-glucopyranosyl)amides indicated the role of specific hydrogen bridges in binding the inhibitors to the enzyme [49].

**Scheme 24:**
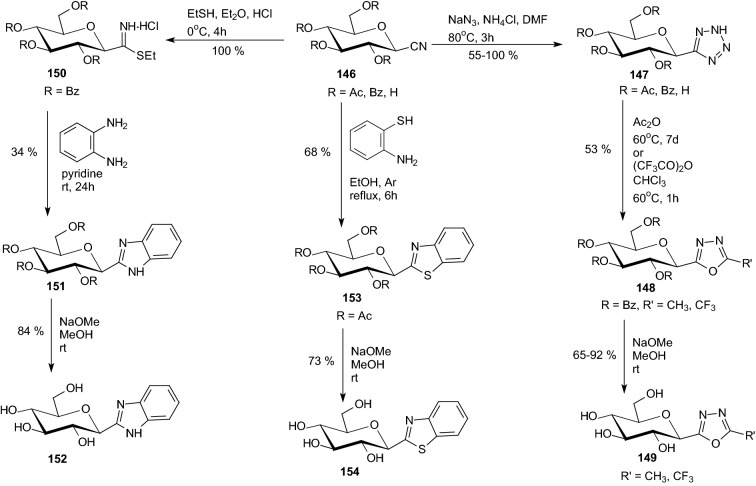
Synthesis of 5-(β-d-glucopyranosyl)tetrazoles **147**, 5-(β-d-glucopyranosyl)-1,3,4-oxadiazoles **149,** 2-(β-d-glucopyranosyl)benzothiazoles **154** and 2-(β-d-glucopyranosyl)-benzimidazoles **152** [50]

In 2004, Somsak et al. have decided to prepare *C*-(β-d-glucopyranosyl) heterocycles exhibiting acidic, basic, and neutral properties in the heterocyclic moieties [50]. They transformed per-*O*-acetylated and -benzoylated β-d-glucopyranosyl cyanides **146** into the corresponding 5-(β-d-glucopyranosyl)tetrazoles **147**, 2-(β-d-glucopyranosyl)benzothiazoles **153** and 2-(β-d-glucopyranosyl)-benzimidazoles **151** (Scheme 24). Acylation of the tetrazoles **147**, either by acetic or trifluoroacetic anhydride, gave 5-(β-d-glucopyranosyl)-2-methyl- and -2-trifluoromethyl-1,3,4-oxadiazoles **148**, respectively. Removal of the protecting groups furnished inhibitors **147** (R=H), **149**, **152**, and **154** exhibiting inhibitor constants in the micromolar range. The tetrazole **147** (R=H) ring of slightly acidic character was unfavorable for the binding of this compound to the GP enzyme. The neutral aglycones in **149** (*K*_i_ = 212 μM) and **154** (*K*_i_ = 229 μM) result in moderate inhibitors. The most efficient inhibitor was benzimidazole **152** (*K*_i_ = 11 μM) [50].

**Fig. 12:**
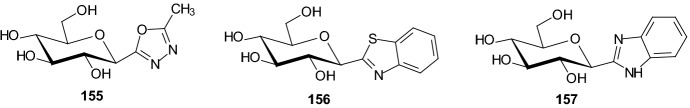
2-(β-d-glucopyranosyl)-5-methyl-1,3,4-oxadiazole **155**, 2-(β-d-glucopyranosyl)-benzothiazole **156** and 2-(β-d-glucopyranosyl)-benzimidazole **157** [51]

In 2005, Chrysina et al. examined inhibitors with enhanced affinity for glycogen phosphorylase that might control hyperglycemia in type 2 diabetes [51]. Three analogs of β-d-glucopyranose: 2-(β-d-glucopyranosyl)-5-methyl-1,3,4-oxadiazole **155**, 2-(β-d-glucopyranosyl)-benzothiazole **156** and 2-(β-d-glucopyranosyl)-benzimidazole **157** were examined (Fig. 12). The compounds showed competitive inhibition with *K*_i_ values of 145.2 μM, 76 μM and 8.6 μM, respectively. In order to establish the mechanism of this inhibition, crystallographic studies were carried out and the structures of GPb in complex with the three analogs were determined. The complex structures revealed that the inhibitors can be accommodated in the catalytic site of T-state GPb with very little change of the tertiary structure [51].

**Scheme 25:**
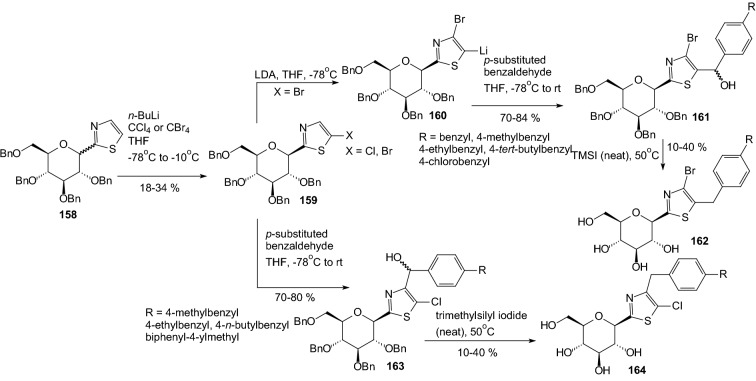
Synthesis of benzylthiazolyl-*C*-glucosides **162** and **164** [52]

**Scheme 26:**
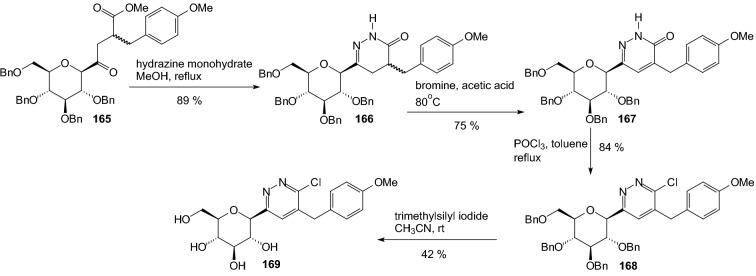
Synthesis of benzylpyridazinyl-*C*-glucosides **169** [52]

In 2010, Kang and coworkers designed and synthesized pyridazinyl and thiazolyl derivative of *C*-glycosides [52]. They wanted to check if replacement of the phenyl ring with the corresponding heterocyclic ring could improve the GLT2 inhibitor. As shown in Scheme 25, the lithiated thiazolylglucoside **158** was converted to 5-chlorothiazolylglucoside or 5-bromothiazolylglucoside **159** by electrophilic halogenation using CCl_4_ and CBr_4_, respectively. Lithiation of 5-bromothiazole intermediate **159** was performed by treatment of LDA, and the resulting anion underwent a metal–halogen exchange reaction so that a bromine atom moved to a new position on the thiazole ring. The lithiated intermediate **160** was subjected to coupling with aldehydes to produce the desired products **161**. The same conditions were applied to 5-chlorothiazole **159**. The chlorine atom did not move to the 4-position but maintained the original position. The coupling reactions of 5-chlorothiazole intermediate **159** with aldehydes produced **163**. Both debenzylation and reduction were concurrently performed to prepare the final products **162** and **164** (Scheme 25). Introduction of the pyridazine ring at the anomeric carbon of d-glucopyranose was carried out in a stereoselective fashion [52]. Cyclization from γ-keto ester **165** to dihydropyridazinone **166** was accomplished with hydrazine monohydrate (Scheme 26). Dihydropyridazinone **166** was oxidized to pyridazinone **167** using bromine under acetic acid. Pyridazinone **167** was converted to 6-chloro-5-benzylpyridazine **168** by treatment with POCl_3_. Final removal of the four benzyl groups to produce the target compound **169** was accomplished with application TMSI (Scheme 26).

Biological activities of the compounds **162**, **164** (Scheme 25) and **169** (Scheme 26) were evaluated by in vitro SGLT2 inhibition assay. While dapagliflozin (Scheme 18) shows highly potent inhibitory activity against human hSGLT2, it was discovered that neither pyridazinyl nor thiazolyl analogs improved hSGLT2 inhibition [52].

**Scheme 27:**
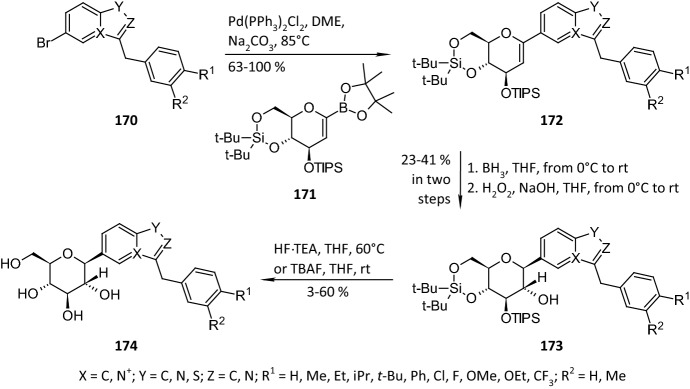
Synthesis of benzisothiazole- and indolizine-*β*-d-glucopyranosides **174** as SGLT2 inhibitors [53]

In 2010, Handlon and coworkers described a method of obtaining *C*-linked heterocyclic glucosides that could inhibit human SGLT2 [53]. The authors used the bromo heterocycles **170** and the glucal boronate **171** to obtain a series of benzisothiazole- and indolizine-β-d-glucopyranosides **174** (Scheme 27). The key step of the reactions was a palladium-catalyzed cross-coupling leading to intermediates **172**. Subsequent hydroboration–oxidation reactions followed by an acidic deprotection of the sugar rings in the molecules of **173** provided the final products **174**. The substrates **170** were obtained in three various ways, depending on their heterocyclic cores. The compounds were evaluated for their human SGLT1 and SGLT2 inhibition potential by monitoring the suppression of the uptake of ^14^C-labeled α-methyl-d-glucopyranoside by COS-7 cells, which transiently expressed human SGLT2 or SGLT1, using BacMam technology [54]. The authors focused mostly on the influence of the character of the substituents R^1^ and R^2^ and the basicity of the aromatic core on the inhibition potential of the compounds. It was found that their oral absorptions were good enough to avoid a transformation into the corresponding pro-drugs prior to the intake. Finally, the compound **174** (X=C, Y=S, Z=N, R^1^=*t*-Bu, R^2^=H) was found to be an inhibitor of SGLT2 with an IC_50_ of 10 nM [53].

**Scheme 28:**
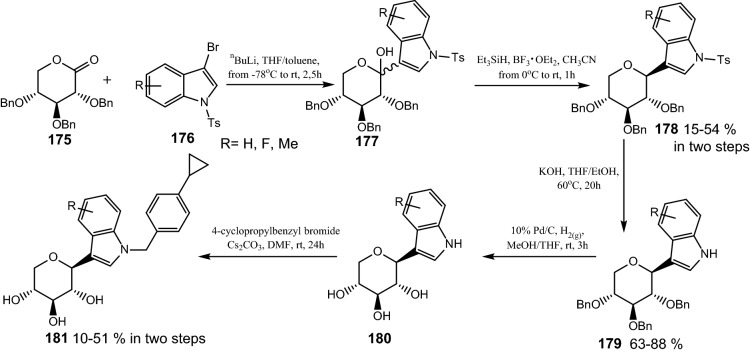
Synthesis of *C*-indolylxylosides **181** bearing *p*-cyklopropylbenzyl group [55]

In 2012, Yao et al. based on previous research into usage of *N*-indolylxylosides as SGLT2 inhibitors [55] and knowledge about metabolic stability of the *C*-glycosidic bond, synthesized the *C*-indolylxylosides as a result of a five-step synthesis. It is noteworthy that their SAR studies disclosed the key role of two substituents in the indole moiety. The presence of both a distal *p*-cyclopropylphenyl group and substituent in 7-position is necessary to achieve potent inhibitory activity. Using 2,3,4-tri-*O*-benzyl-d-xylonolactone **175** and diverse 3-bromo-1-tosyl-1*H*-indoles **176** as a starting material in lithium halogen exchange reaction, a variety of lactols **177** were received. Reduction with trietylsilane and boron trifluoride etherate gave *C*-linked β-xylosides **178** (Scheme 28). During heating of the previously obtained compounds **178** over KOH in THF/EtOH, detosylation took place, providing free indoles **179**. Benzyl ether groups of **179** were removed under hydrogenolysis to furnish **180**. Xylopyranosyl indoles **180** underwent *N*-alkylation with *p*-cyclopropylbenzyl bromide and gave the final products **181**. Evaluation of biological activity demonstrated that from among *C*-indolylxylosides, compound **181** (R=F) turned out to be the strongest and metabolically stable SGLT2 and SGLT1 inhibitor. In compliance with SAR studies bearing two groups most significant for inhibition activity, it exhibits an SGLT2 EC_50_ value of 47 nM and SGLT1 EC_50_ value of 282 nM. Moreover pharmacokinetic studies showed that molecule **181** (R=7-F) is metabolically stable after intravenous and oral administration to rats [55].

**Scheme 29:**
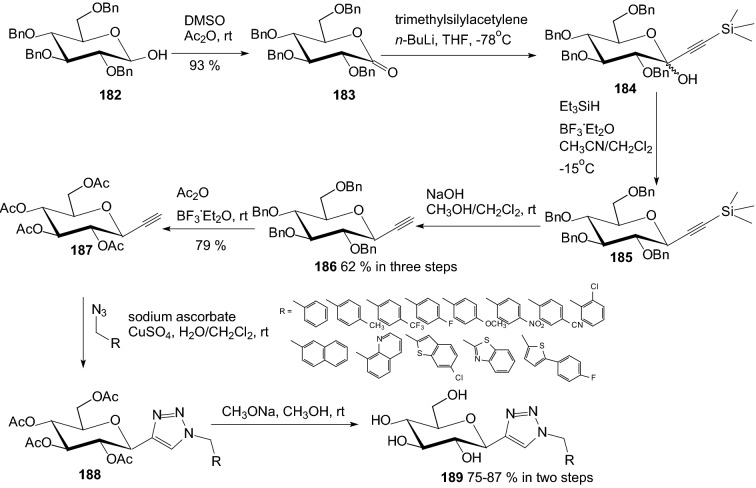
Synthesis of *C*-glucosides with triazole aglycone **189** [56]

In 2012 Li et al. designed and synthesized analogs of SGLT2 inhibitors containing the 1,2,3-triazole motif [56]. Substituted 1,2,3-triazole is a very important building block for more complex bioactive compounds, such as tazobactam, antiviral, anti-HIV, antibacterial, and antiallergic agents [56]. The *C*-glucosides with triazole aglycone were constructed by click chemistry. The synthesis of the key alkyne intermediate is outlined in Scheme 29. Alkyne **187** was obtained from 2,3,4,6-tetra-*O*-benzyl-d-glucopyranose **182** in five steps. 2,3,4,6-Tetra-O-benzyl-d-(+)-glucono-1,5-lactone **183** was prepared by Swern oxidation of benzyl protected d-glucopyranose **182**. Trimethylsilylacetylene was deprotonated with *n*-BuLi and treated with lactone **183** to provide ketose **184**. The free hydroxyl group was reduced and the trimethylsilyl group was removed easily by stirring in a mixture of NaOH, methanol, and dichloromethane, yielding the benzyl-protected alkyne **186**. Alkyne **186** and azides were used directly to construct triazole aglycon by click chemistry. Compound **186** was transformed into the acetyl-protected form **187**. Triazoles **188** were then synthesized through CuAAC with the corresponding azides (Scheme 29). Finally, the acetyl protecting groups were removed to give the triazole-linked *C*-glycosides compounds **189**. Most of the synthesized compounds demonstrated increased urinary glucose excretion in SD rats, but they increased urine volume to a lesser degree than that of dapagliflozin [56].

**Scheme 30:**
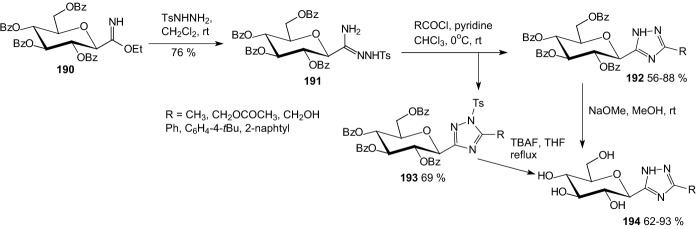
Synthesis of 3-(β-d-glucopyranosyl)-5-substituted-1,2,4-triazoles **194** [57]

In 2013, Bokor et al. elaborated a new method for the synthesis of 3-(β-d-glucopyranosyl)-5-substituted-1,2,4-triazoles [57]. The starting compound was *O*-perbenzoylated β-d-glucopyranosyl formimidate **190**, which reacted with tosylhydrazide to give tosylamidrazone **191** (Scheme 30). 3-(β-d-Glucopyranosyl)-5-substituted-1,2,4-triazoles **194** were prepared by acylation of *O*-perbenzoylated *N*1-tosyl-*C*-β-d-glucopyranosyl formamidrazone **191** and subsequent removal of the protecting groups. The best inhibitor was 3-(β-d-glucopyranosyl)-5-(2-naphthyl)-1,2,4-triazole **194** (*K*_i_ = 0.41 μM against rabbit muscle glycogen phosphorylase b).

**Scheme 31:**
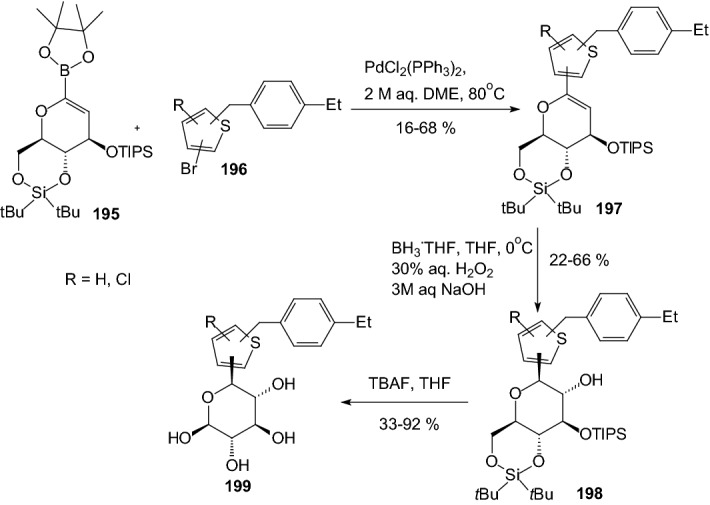
Synthesis of thiophene-*C*-glucosides **199** [58]

In 2013, Sakamaki and coworkers described the synthesis and structure–activity relationship of thiophene-*C*-glucosides [58]. The synthetic route to thiophene-*C*-glucosides **199** is shown in Scheme 31, based on the reaction of aryl halide **196** with glucal-boronate ester **195**. Coupling reaction with using dichlorobis (triphenylphosphine) palladium between aglycones **195** and glucal-boronate **196** gave **197**, followed by stereoselective hydroboration and oxidation in alkaline conditions yielded **198** with the desired β-configuration. *O*-silyl groups of **198** were deprotected with tetra-*n*-butylammonium fluoride (TBAF) to afford thiophene-*C*-glucosides **199** (Scheme 31). The human hSGLT2 inhibitory activities and rat urinary glucose excretion (UGE) effects of **199** were evaluated. As a result, they showed good hSGLT2 inhibitory activities. In particular, the chlorothiophene derivative **199** showed remarkable inhibitory activity against hSGLT2 (IC_50_ = 4.0 nM) [58].

**Scheme 32:**
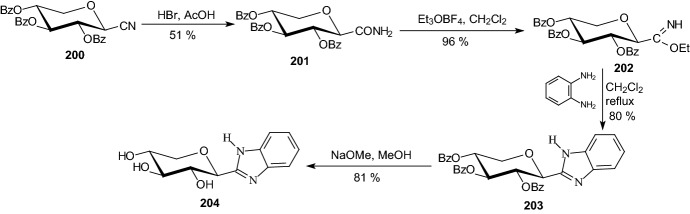
Synthesis of 2-(β-d-xylopyranosyl)benzimidazole **204** [59**]**

**Scheme 33:**
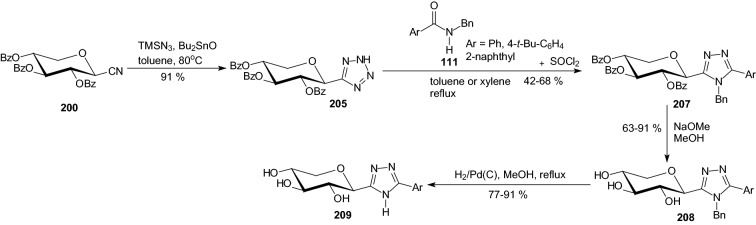
Synthesis of 3-(β-d-xylopyranosyl)-5-substituted-1,2,4-triazoles **209** [59**]**

In 2014, Somsak et al. synthesized derivatives of d-xylose with aglycones of the most efficient glucose-derived inhibitors of glycogen phosphorylase to explore the specificity of the enzyme towards the structure of the sugar part of the molecules [59]. 2-(β-d-Xylopyranosyl)benzimidazole **204** (Scheme 32) and 3-substituted-5-(β-d-xylopyranosyl)-1,2,4-triazoles **209** (Scheme 33) were obtained in multistep procedures from *O*-perbenzoylated β-d-xylopyranosyl cyanide **200**.

**Scheme 34:**
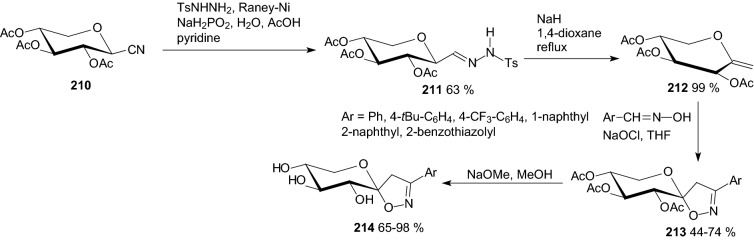
Synthesis of xylopyranosylidene-spiro-isoxazoline derivatives **214** [59**]**

Cycloadditions of nitrile-oxides and *O*-peracetylated exo-xylal **212** obtained from the corresponding β-d-xylopyranosyl cyanide **210** furnished xylopyranosylidene-spiro-isoxazoline derivatives **214** (Scheme 34) [59].

**Scheme 35:**
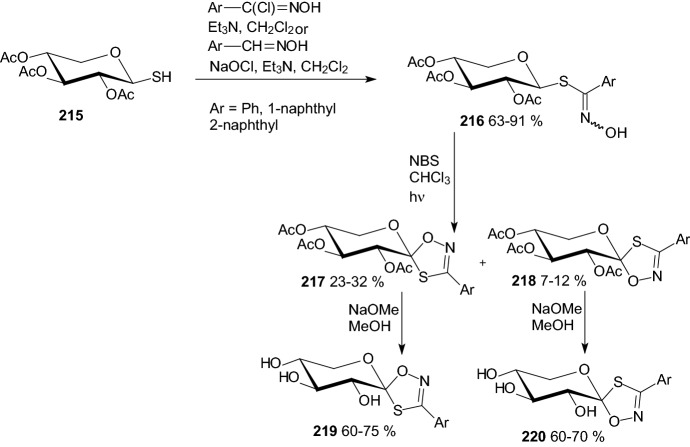
Synthesis of xylopyranosylidene-spiro-oxathiazoles **219** and **220** [59**]**

Oxidative ring closure of *O*-peracetylated β-d-xylopyranosyl-thiohydroximates prepared from 1-thio-β-d-xylopyranose **215** and nitrile-oxides gave xylopyranosylidene-spiro-oxathiazoles **217** and **218** (Scheme 35) [59]. The fully deprotected test compounds **204**, **209**, **214, 219**, and **220** were assayed against rabbit muscle glycogen phosphorylase *b*. Evaluation showed very weak inhibition for 3-(2-naphthyl)-5-(β-d-xylopyranosyl)-1,2,4-triazole **209** only, while all other compounds proved ineffective in a concentration of 625 μM. Observations showed that the aglycones rendering their glucose derivatives to nanomolar inhibitors are not yet capable of completely overriding the effect of losing the side chain of the glucose moiety [59].

**Scheme 36:**
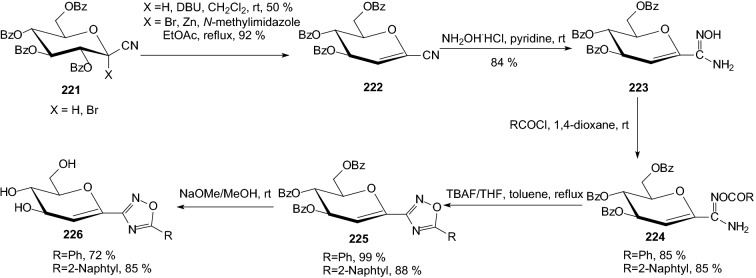
First method of compounds **226** synthesis [62]

**Scheme 37:**
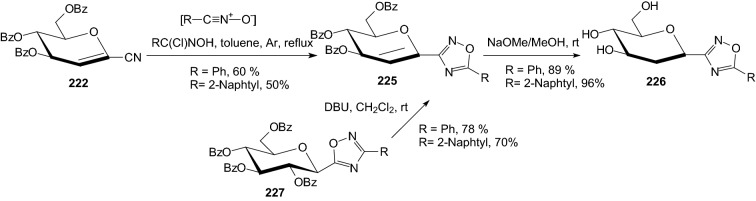
Second method of compounds **226** synthesis [62]

Investigations on the inhibitory and binding properties of different monosaccharides indicated the superior effectiveness of d-glucose [60, 61]. Changes in the sugar configuration as well as removal or replacement of substituents of the glucose moiety proved detrimental for the inhibition. Therefore, in 2015 Bokor et al. elaborated synthetic methods for *D*-glucal attached to oxadiazoles by a C–C bond [62]. For the preparation of the target compounds **226**, two main routes were used; the functionalized glucal **226** was made by the formation of the heterocycle in the final stage (Scheme 36) or the 1,2-double bond can be introduced into a preformed *C*-glucopyranosyl heterocycle **227** (Scheme 37). Introduction of the double bond was effected by either DBU induced elimination of benzoic acid from *O*-perbenzoylated glucopyranosyl precursors **221** (X=H) or Zn/N-methylimidazole mediated reductive elimination from the 1-bromoglucopyranosyl starting compounds **221** (X=Br) (Scheme 36). Test compounds **226** were obtained by Zemplen debenzoylation. Unfortunately, none of these showed significant inhibition of rabbit muscle glycogen phosphorylase b, indicating that the binding of the aglycones was not strong enough to override the detrimental effects of the changes in the sugar parts of the molecules [62].

**Scheme 38:**
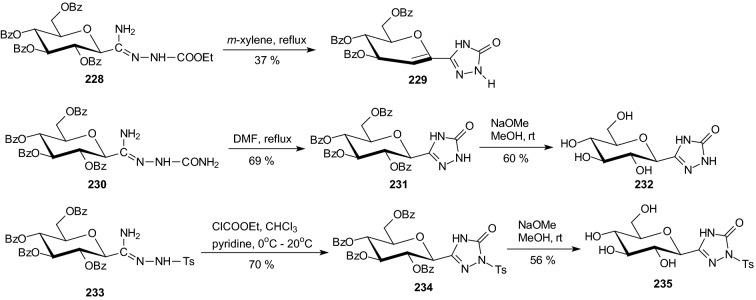
Syntheses of *C*-glucopyranosyl-1,2,4-triazolones **229**, **232**, and **235** [63]

**Scheme 39:**
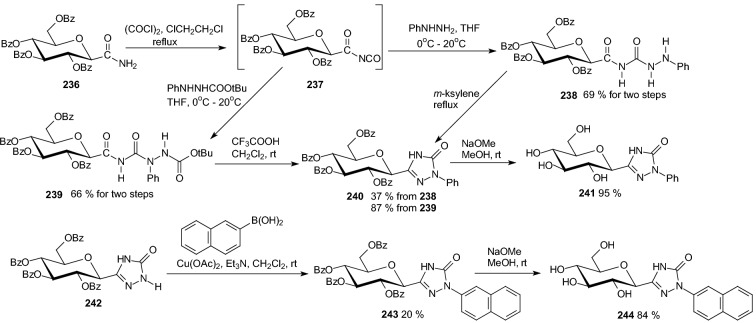
Synthesis of 3-β-D-glucopyranosyl-1-phenyl-1,2,4-triazol-5-one **241** and 1-(2-naphthyl) derivative **244** [63]

In 2016, Bokor et al. designed various *C*-glucopyranosyl-1,2,4-triazolones as potential inhibitors of GP [63]. Syntheses of these compounds were performed with *O*-perbenzoylated glucose derivatives **228**, **230**, and **233** as precursors (Scheme 38). Boiling a solution of carbamoyl-*C*-β-d-glucopyranosyl formamidrazone **228** in *m*-xylene gave 3-β-d-glucopyranosyl-1,2,4-triazol-5-one **229**. Cyclization of **230** in boiling DMF produced the expected triazolone **231**. Reaction of tosyl-*C*-β-d-glucopyranosyl formamidrazone **233** with ethyl chloroformate furnished 3-β-d-glucopyranosyl-1-tosyl-1,2,4-triazol-5-one **234** (Scheme 38). In situ prepared β-d-glucopyranosylcarbonyl isocyanate **237** was transformed by PhNHNHBoc into 3-β-d-glucopyranosyl-1-phenyl-1,2,4-triazol-5-one **240**, while the analogous 1-(2-naphthyl) derivative **243** was obtained from the unsubstituted triazolone **242** by naphthalene-2-boronic acid in a Cu(II) catalyzed *N*-arylation (Scheme 39). Test compounds were prepared by Zemplen deacylation. The new glucose derivatives had weak or no inhibition of rabbit muscle glycogen phosphorylase b. The best inhibitor was 3-β-d-glucopyranosyl-1-(2-naphthyl)-1,2,4-triazol-5-one **244** (*K*_i_ = 80 μM) (Scheme 39) [63].

**Scheme 40:**
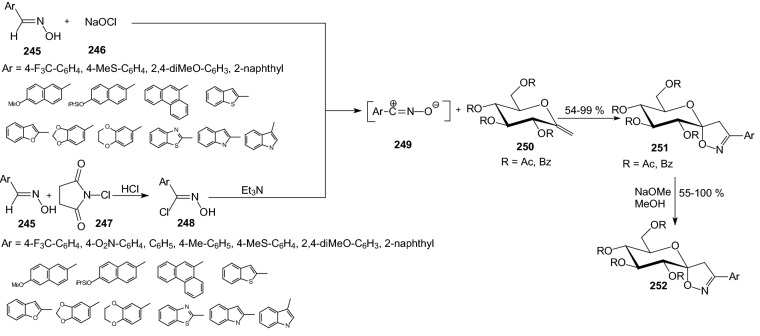
Synthesis of spiro-isooxazolines **252** by 1,3-dipolar cycloaddition of nitrile oxides **249** to exo-glucals **250** [64]

Glucose-based spiro-isoxazolines can be considered as anti-hyperglycemic agents against type 2 diabetes through GP inhibition. In 2016, d-glucopyranosylidene-spiro-isoxazolines **252** were prepared by 1,3-dipolar cycloaddition of nitrile oxides **249** generated in situ to methylene exo-glucals **250** (Scheme 40) [64]. Reagents **249** were generated by reaction of a sodium hypochlorite **246** and oximes **245**. Appropriate oximes **245** reacted also with NCS **247** and aryl α-chloroaldoximes **248** were prepared. Hydrochloric acid elimination in the presence of NEt_3_ afforded reactive nitrile oxides **249**. *O*-unprotected spiro-isooxazolines **252** were evaluated as GP inhibitors and exhibited IC_50_ values ranging from 1 to 800 μM. The tetra-*O*-acetylated spiro-isoxazoline **251** bearing 2-naphthyl residue shoved a much lower value compared to that of the *O*-unprotected analog **252** [64]. The 2-naphthyl substituted glucopyranosylidene-spiro-isoxazoline **252** was the best compound identified in this study (GPb *K*_i_ = 0.63 μM).

**Scheme 41:**
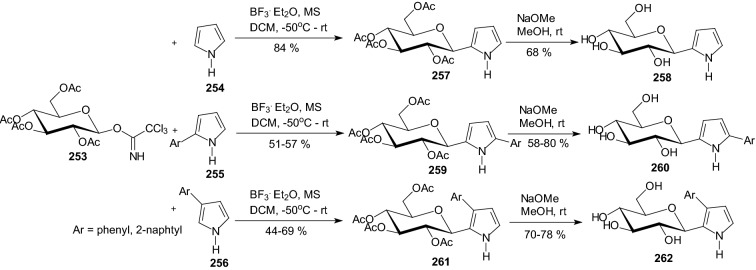
Synthesis of *C*-β-d-glucopyranosyl pyrrole derivatives **258**, **260**, and **262** [65]

**Scheme 42:**
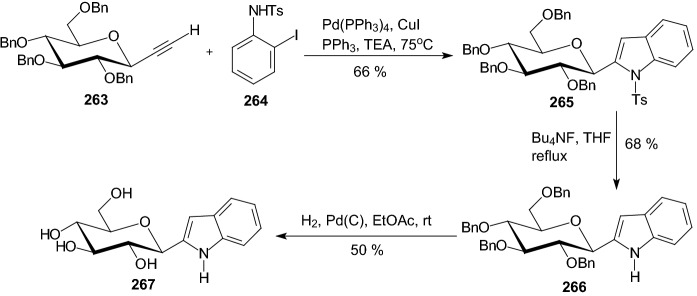
Synthesis of 2-(β-d-glucopyranosyl) indole **267** [65]

**Scheme 43:**
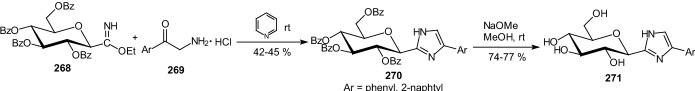
Synthesis of 2-(β-d-glucopyranosyl) imidazoles **271** [65]

Syntheses of a series of *C*-glucopyranosyl pyrroles, indole, and an improved preparation of *C*-glucopyranosyl imidazoles allowed in 2016 Kantsadi et al. to study and compare their inhibitory efficiency against GP [65]. *C*-β-D-Glucopyranosyl pyrrole derivatives **258**, **260**, and **262** were prepared in the reactions of pyrrole **254**, 2-aryl-pyroles **255**, and 3-aryl-pyrroles **256** with *O*-peracetylated β-d-glucopyranosyl trichloroacetimidate **253** (Scheme 41). (2β-d-Glucopyranosyl) indole **267** was obtained by a cross-coupling of *O*-perbenzylated β-d-glucopyranosyl acetylene **263** with *N*-tosyl-2-iodoaniline **264** followed by spontaneous ring closure (Scheme 42) [65]. An improved synthesis of *O*-perbenzoylated 2-(β-d-glucopyranosyl) imidazoles **270** was achieved by reacting *C*-glucopyranosyl formimidates **268** with α-aminoketones **269** (Scheme 43) [65]. The deprotected compounds were assayed with isoforms of glycogen phosphorylase to show no activity of the pyrroles **258**, **260**, **262**, and indole **267** against rabbit muscle GPb [65]. The imidazoles **271** proved to be the best-known glucose-derived inhibitors of not only the muscle enzymes (both a and b) but also of the pharmacologically relevant human liver hlGPa (*K*_i_ = 156 and 26 nM for the phenyl and 2-naphthyl derivatives, respectively). An X-ray crystallographic study of the rmGPb-imidazole complexes revealed structural features of the strong binding, and also allowed explaining the absence of inhibition for the pyrrole and indole derivatives [65].

**Fig. 13:**
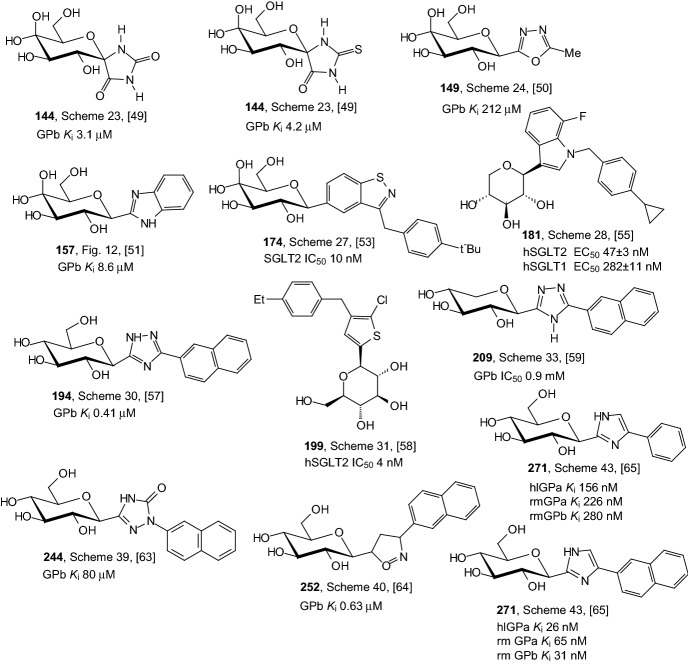
Inhibitory properties of the best inhibitors from the heteroaromatic *C*-glycosyl derivatives described in Sect. 4.2

Figure 13 shows the structure of the best inhibitors from heteroaromatic *C*-glycoside derivatives described in Sect. 4.2. Again, the analysis of the structure of the best inhibitors leads to the conclusion that the highest activity is ensured by the presence of glucose as a structural element. Only in the case of compounds **181** and **209** is it xylose. It can also be seen that the high inhibitor activity is guaranteed by the presence of a structural element such as a five-membered heteroaromatic ring containing two or three nitrogen atoms. The high activity of the inhibitor is also ensured by the presence of such heteroaromatic rings as: 1,3,4-oxadiazole (compound **149**), benzisothiazole (compound **174**), indole (compound **181**), thiophene (compound **199**), and isoxazoline (compound **252**). Again, presents a distal *p*-cyclopropylphenyl group (compound **181**) is necessary to achieve potent inhibitory activity. Also, the glycone and aglycone spiro combination (compounds **144**) ensures high inhibitor activity.

### Other *C*-Glycosyl Derivatives

**Scheme 44:**
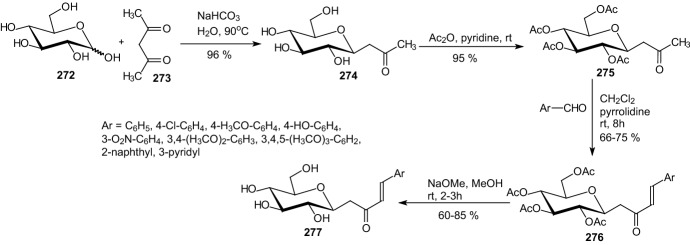
Synthesis of (*E*)-4-aryl-1-(β-d-glucopyranosyl)-but-3-en-2-ones **277** [66]

In 2009, Bisht and coworkers described the synthesis of aryl butenoyl *C*-glycosides **277** by aldol condensation of peracetylated glycosyl acetones **275** with aromatic aldehydes followed by deacetylation with methanolic NaOMe (Scheme 44) [66]. β-*C*-Glycosidic ketones **274** were prepared in one step directly from the unprotected sugar **272** and pentane-2,4-dione **273** under aqueous conditions by Knoevenagel condensation [67]. Compounds **275** on aldol reaction with different aldehydes under ambient reaction conditions resulted in (*E*)-4-aryl-1-(glycopyranosyl)-but-3-en-2-ones **276** [68]. Prepared *C*-glycosides **277** were evaluated for their α-glucosidase, glucose-6-phosphatse, and glycogen phosphorylase enzyme inhibitory activities in vitro and in vivo. Three of the compounds **277** (Ar=2-naphthyl, phenyl, 3,4-dimethoxyphenyl) showed potent enzyme inhibitory activities as compared to standard drugs such as acarbose and metformin. These *C*-glycosides caused a significant decline in the hyperglycemia of the diabetic rats post sucrose-load [66].

**Scheme 45:**
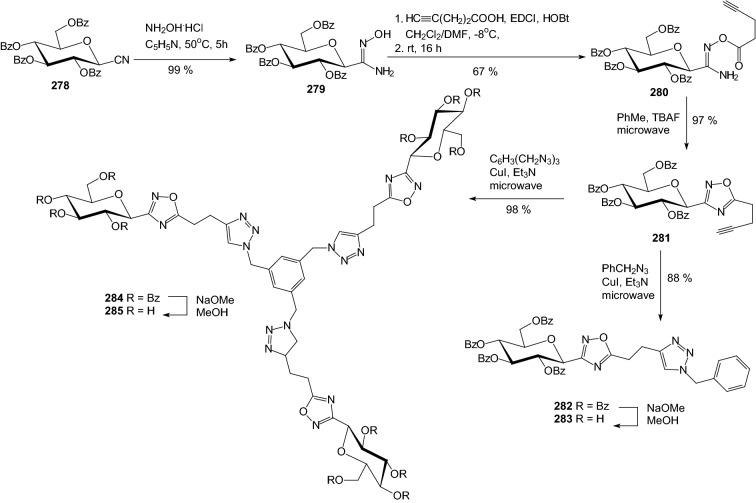
Synthesis of inhibitors of GP **283** and **285** through Cu(I)-assisted 1,3-dipolar cycloaddition [69]

Another approach for the inhibition of GP could take advantage of multivalency. In 2009, Cecioni and coworkers examined influence multivalency for the inhibition of GP [69]. They synthesized two distinct trivalent inhibitors of GP through Cu(I)-assisted 1,3-dipolar cycloaddition and by formation of a trisoxadiazole derivative. The perbenzoylated glucosyl cyanide **278** was reacted with hydroxylamine hydrochloride in pyridine to afford the desired amidoxime **279** (Scheme 45). The formation of the *O*-acyl-amidoxime **280** was achieved with 4-pentynoic acid in the presence of EDCI/HOBt as coupling agents. The use of thermal activation combined with TBAF catalysis provided the cyclic oxadiazole **281**. The alkyne-terminated oxadiazole **281** was then engaged in a Huisgen’s Cu(I)-catalyzed 1,3-dipolar cycloaddition reaction under microwaves activation with benzyl azide to afford 1,4-disubstituted 1,2,3-triazole **282**. Debenzoylation of compound **282** afforded hydroxylated GP inhibitor candidate **283**. The reaction of 1,3,5-tris(azidomethyl)benzene with the alkyne derivative **281** under microwave activation and Cu(I) catalysis afforded the cycloadduct **284**. The saponification of the benzoate ester **284** provided the fully hydroxylated macromolecule **285**. Also, a more condensed trifunctional macromolecule was prepared in which the *C*-glucosyl-oxadiazole moiety was directly attached to a benzene ring. A biological study of the inhibiting properties of these trivalent inhibitors of GP have shown that the valency of the molecules influences slightly the inhibition of the enzyme, whereas the presence of a spacer arm between the core and the pharmacophore moieties does not. Authors reassumed that multivalent inhibitors were always superior to their monovalent counterparts [69].

**Scheme 46:**
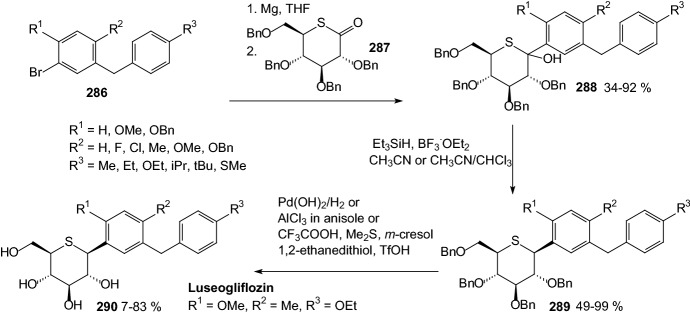
Synthesis of *C*-phenyl 1-thio-d-glucitols **290** selective SGLT2 inhibitors [70]

In 2010, Kakinuma et al. gave considerable attention to 5-thioglucose—derived SGLT2 inhibitors, which have a sulfur atom in place of the oxygen atom in the glucose ring [70]. It is known that 5′-thio-*N*-acetyllactosamine is 200 times more resistant to digestion by β-galactosidase [71], and methyl α-5′-thiomaltoside is not hydrolyzed at all by glucoamylase [72]. In previous articles [73, 74], it was also described that *O*-aryl 5-thio-β-glucoside is a SGLT inhibitor. Kakinuma et al. developed a synthetic strategy for preparing *C*-phenyl 1-thio-d-glucitol derivatives **290**, as it is outlined in Scheme 46 [70]. Compounds **288** were obtained by adding thiolactone **287** to Grignard reagents prepared from compounds **286** and magnesium powder. The hydroxyl group of **288** was reduced β-stereoselectively to afford compounds **289**. Finally, the benzyl ether of compounds **289** was removed by catalytic hydrogenation with palladium hydroxide under a hydrogen atmosphere or, alternatively, compounds **290** were obtained by removal of the benzyl group using Lewis acid conditions to prevent reduction of the chloride (Scheme 46). (1*S*)-1,5-Anhydro-1-[5-(4-ethoxybenzyl)-2-methoxy-4-methylphenyl]-1-thio-d-glucitol **290** (R^1^=OMe, R^2^=Me, R^3^=OEt) exhibited potent SGLT2 inhibition activity (IC_50_ = 2.26 nM) [70]. Since 2014, this 1-thio-d-glucitol **290** has been known as luseogliflozin, and it is an orally active SGLT2 inhibitor developed by Taisho Pharmaceutical for the treatment of patients with type 2 diabetes mellitus [75].

**Fig. 14:**
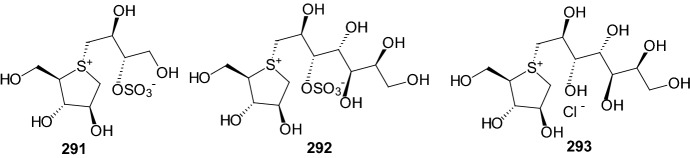
Structures of R-glucosidase inhibitors from* Salacia reticulata*: salacinol **291**, kotalanol **292**, and de-*O*-sulfonated kotalanol **293** [76]

An approach to controlling blood glucose levels in individuals with type 2 diabetes is to target *R*-amylases and intestinal glucosidases using *R*-glucosidase inhibitors acarbose and miglitol. One of the intestinal glucosidases targeted is the N-terminal catalytic domain of maltase-glucoamylase (ntMGAM), one of the four intestinal glycoside hydrolase 31 enzyme activities responsible for the hydrolysis of terminal starch products into glucose [76]. In 2010, Sim and coworkers presented the X-ray crystallographic studies of ntMGAM in complex with a new class of R-glucosidase inhibitors derived from natural extracts of* Salacia reticulata*, a plant used traditionally in Ayurvedic medicine for the treatment of type 2 diabetes [76]. In extracts, active compounds were: salacinol **291**, kotalanol **292**, and de-*O*-sulfonated kotalanol **293** (Fig. 14). This study revealed that kotalanol **293** is the most potent ntMGAM inhibitor reported to date (*K*_i_ = 0.03 μM), some 2000-fold better than the compounds currently used in the clinic, and highlights the potential of the salacinol class of inhibitors as future drug candidates [76].

**Scheme 47:**
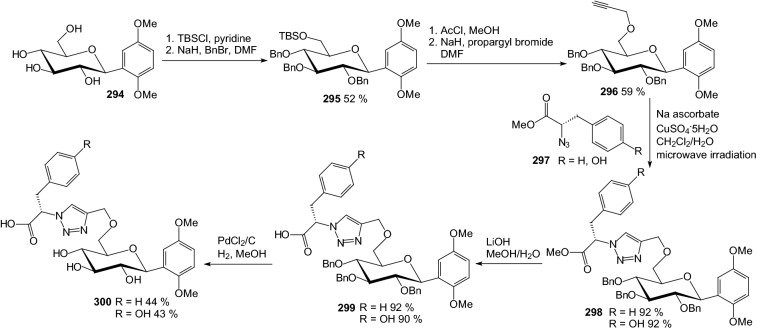
Synthesis of triazolyl phenylalanine and tyrosine-aryl *C*-glycoside hybrids **300** via microwave-assisted Cu(I)-catalyzed azide-alkyne 1,3-dipolar cycloaddition [77]

In 2011, Wang et al. synthesized triazolyl phenylalanine and tyrosine-aryl *C*-glycoside hybrids via microwave-assisted Cu(I)-catalyzed azide-alkyne 1,3-dipolar cycloaddition [77]. Successive enzymatic assay identified the synthesized glycoconjugates as novel PTP1B inhibitors with low micromole-ranged inhibitory activity and at least several-fold selectivity over other homologous PTPs tested. As shown in Scheme 47, the azido phenylalaninyl or tyrosinyl derivatives **297** were synthesized according to the literature [78]. For the synthesis of the *O*-propynyl *C*-glycoside **296**, the known *C*-glucosyl 1,4-dimethoxybenzene **294** was first regioselectively silylated on its 6-position with TBDMSCl followed by full *O*-benzylation with NaH and BnBr. Then, the TBS group was desilylated with AcCl to give the free 6-OH, which was propargylated in the presence of NaH and propargyl bromide. Huisgen [3 + 2] cycloaddition between the azides **297** and the sugar alkyne **296** was catalyzed by sodium ascorbate and CuSO_4_·5H_2_O yielding the click adducts **298** (Scheme 47). The saponification with LiOH led to the carboxylic acids **299**. The following hydrogenolysis gave the fully deprotected amino acid-sugar hybrid **300**. Benzyl groups on glucosyl moiety of compounds were found crucial for PTP1B inhibition. The biological assay identified the glycoconjugates that contain the carboxylic acid and benzyl moieties as more active PTP1B inhibitors compared to their ester and debenzylated counterparts [77].

**Scheme 48:**
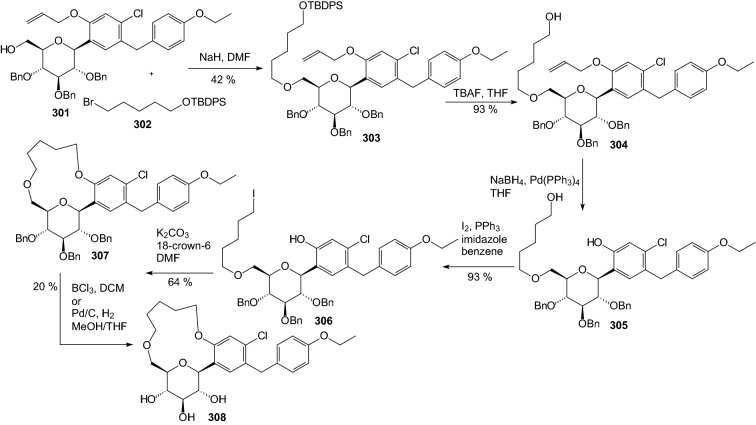
Synthesis of macrocyclic *C*-aryl glucoside SGLT2 inhibitor **308** [79]

In 2011, Kim et al. designed and synthesized novel macrocyclic *C*-aryl glucoside SGLT2 inhibitors [79]. Two different synthetic routes of macrocyclization were adopted. Alkylation of alcohol **301** with (5-bromopentyloxy)(*tert*-butyl)-diphenylsilane **302** in the presence of sodium hydride in DMF produced **303** (Scheme 48). Desilylation of **303** with TBAF gave alcohol **304**. Removal of the allyl group was carried out using NaBH_4_ in the presence of tetrakis(triphenylphosphine) palladium(0) to give phenol **305** in quantitative yield. The primary alcohol of **305** was transformed into the corresponding iodide **306** by action of iodine, triphenylphosphine, and imidazole in benzene. The iodide **306** underwent macrocyclization to **307** under conditions of potassium carbonate and 18-crown-6 in DMF. Removal of the benzyl groups on the carbohydrate moiety proceeded with either BCl_3_ in methylene chloride or hydrogenolysis on Pd/C in MeOH and THF to produce the target compound **308** (Scheme 48). Among the compounds tested, [1, 7] dioxacyclopentadecine macrocycles **308** possessing ethoxyphenyl at the distal ring showed the best in vitro inhibitory activity (IC_50_ = 0.778 nM) against human hSGLT2 [79].

**Scheme 49:**
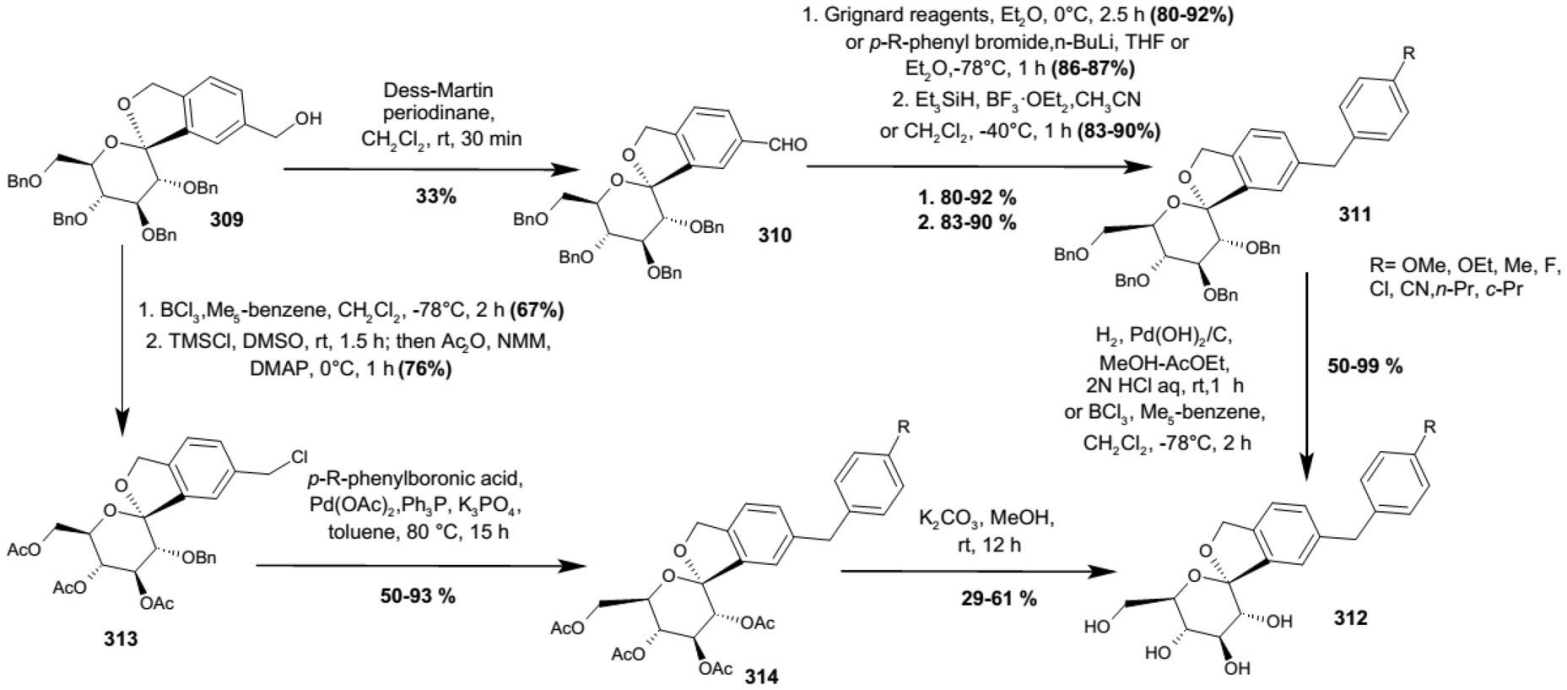
Synthesis of *O*-spiroketal *C*-arylglucoside scaffold **312** [80]

In 2012, Ohtake et al. discovered a novel class of inhibitors, which have an *O*-spiroketal *C*-arylglucoside scaffold [80]. Compound **312** (R^1^=Et)—tofogliflozin (Scheme 49) is a selective SGLT2 inhibitor that is one of the inhibitors for the treatment of type 2 diabetes. Ohtake et al. worked on synthesis of tofogliflozin using the computational modeling and comparing other pharmacophore models that were derived from earlier inhibitors. *O*-Spiroketal *C*-arylglucosides **312** were prepared from **309** through two pathways, as outlined in Scheme 49. Compounds **311** were synthesized from the aldehyde **310**, which could be obtained by oxidation of **309**, followed by addition of Grignard reagents or lithiated benzenes and reduction. Compounds **314** were prepared utilizing the Suzuki coupling reactions. After debenzylation of **309** using boron trichloride, benzyl alcohol moiety was selectively chlorinated by treatment of chlorotrimethylsilane with dimethyl sulfoxide. Four hydroxyl groups of the resulting benzyl chloride were acetylated to afford **313**. Suzuki coupling reactions of **313** with the corresponding 4-substituted phenylboronic acids gave **314**. Deprotections (debenzylation for **311** or deacetylation for **314**) afforded the test compounds **312**. Two products **312** (R=Et or *i*Pr) were submitted to clinical trials. Both products showed a similar degree of increase in renal glucose excretion after oral dosing [80]. However, the next clinical trials turned out the **312** (R=Et) is much better, because it had more desirable profiles in oral bioavailability and renal excretion than **314** (R=*i*Pr).

**Scheme 50:**
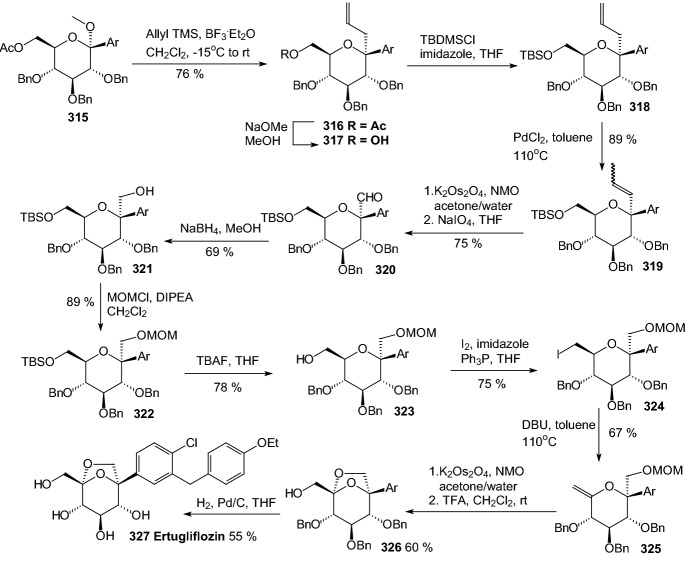
Synthesis of ertugliflozin **327** [81]

The discovery of structurally distinct SGLT2 inhibitors has been mainly focused on the modification of the aglycones, while modification to the glucose residue is less known. Therefore, in 2016 Yan and coworkers decided to examine a series of *C*-aryl glucosides containing dioxa-bicycle for inhibition activity against hSGLT2 [81]. Ertugliflozin [82], bearing a unique dioxa-bicycle in place of the glucose residue of dapagliflozin, is distinct from other inhibitors and shows even better SGLT2 inhibitory activity, which is currently under phase III clinical trial. The synthesis of dioxa-bicycle *C*-aryl glucoside **327** is outlined in Scheme 50. Allylation of **315** in the presence of boron trifluoride etherate formed compound **316**, which was converted to ether **318**. Conversion of the allyl intermediate **318** to aldehyde **320** using a Pd-catalyzed double-bond migration and next reaction with K_2_Os_2_O_4_ and sodium periodate was made. Aldehyde **320** was then reduced to the alcohol **321**, which was then protected as the methoxymethyl ether **322**. Deprotection of TBSO ether gave alcohol **323**, and the primary hydroxyl group of **323** was subjected to iodination using Ph_3_P, imidazole and iodine to give **324**, which upon elimination using DBU in toluene furnished **325**. Sharpless dihydroxylation and acid-promoted one-pot MOM removal followed by stereoselective intramolecular trapping of the putative oxonium ion intermediate gave compound **326**. Hydrogenolysis of the benzyl-protecting groups yielded target compound **327** (Scheme 50). The target compound **327** was subsequently subjected to biological evaluation as novel *C*-aryl glucoside SGLT2 inhibitor. Compound **327** showed good inhibitory activity against *h*SGLT2 IC_50_ = 714 nM [81].

**Scheme 51:**
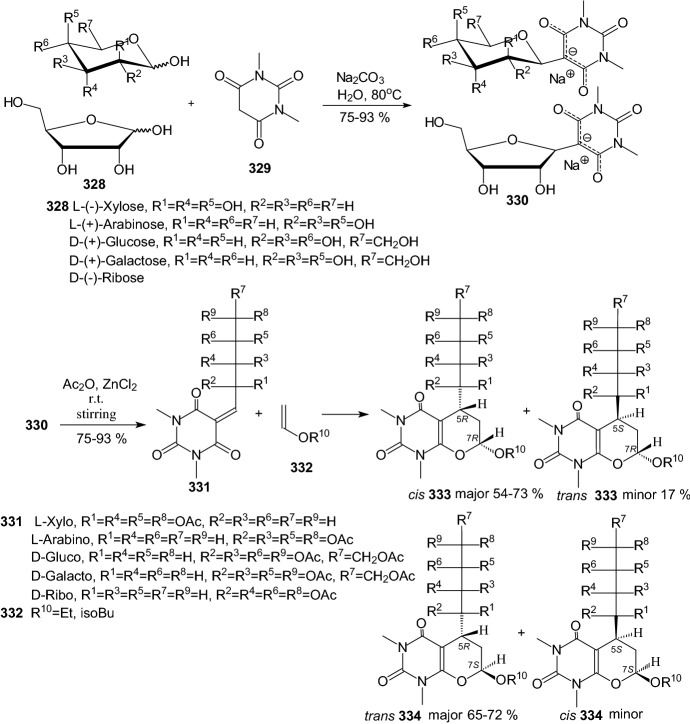
Synthesis of pyrano[2,3-*d*]pyrimidines **333** and **334** with sugar moiety [83]

**Scheme 52:**
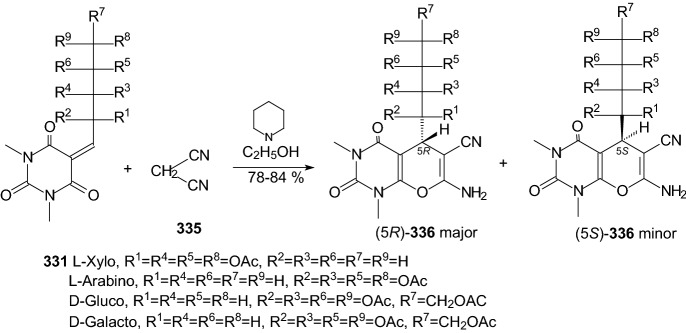
Synthesis of pyrano[2,3-*d*]pyrimidine-6-carbonitriles **336** with a sugar moiety [83]

According to our group interest of synthesis of *C*-glycosyl derivatives we also occupied in the synthesis of some derivatives. In 2013, we developed a convenient and efficient procedure for the preparation of fused uracils - pyrano[2,3-*d*]pyrimidines with sugar moiety [83]. The reaction sequence was: Knoevenagel condensation of unprotected sugars **328** and 1,3-dimethylbarbituric acid **329** in water, acetylation of *C*-glycosides **330** and hetero-Diels–Alder reaction (Scheme 51). *O*-Acetylated 1,3-dimethyl-2,4,6-trioxo-pyrimidin-5-ylidene derivatives **331** were used as new heterodienes in the synthesis of pyrano[2,3-*d*]pyrimidines **333** and **334** containing a sugar moiety. Solvent-free hetero-Diels–Alder cycloadditions of *O*-acetylated pyrimidin-5-ylidene alditols **331** with enol ethers **332** were investigated at room temperature. New, enantiomerically pure *cis* and *trans* diastereoisomers of pyrano[2,3-*d*]pyrimidines **333** with alditol moiety were obtained. The same pyrimidin-5-ylidene alditols **331** underwent conjugate Michael addition-cyclizations with malononitrile **335** at room temperature to afford optically active uracils **336**—diastereoisomers of pyrano[2,3-*d*]pyrimidine-6-carbonitriles with a sugar moiety (Scheme 52) [83]. None of the *C*-glycosyl derivatives of pyrano[2,3-*d*]pyrimidines presented in Schemes 51 and 52 have been evaluated for their pharmacological activity as inhibitors in treatment of type 2 diabetes mellitus.

**Scheme 53:**
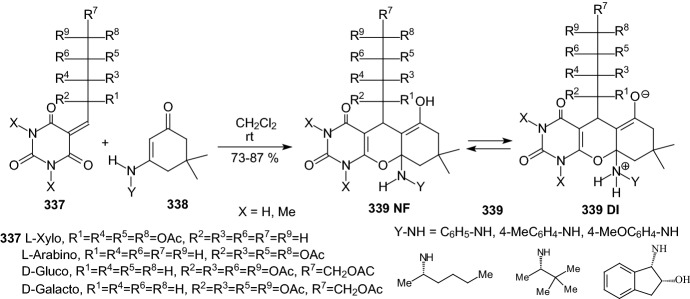
Synthesis of *C*-glycosides-chromeno[2,3-*d*]pyrimidines **339** containing different sugar moieties [84]

Our group also described a convenient and efficient method for the synthesis of chromeno[2,3-*d*]pyrimidine-2,4-diones containing different sugar moieties [84]. Dimedone enamines were used as dienophiles in hetero-Diels–Alder reactions. The cycloaddition reactions of *O*-acetylated 1,3-dimethyl-2,4,6-trioxo-pyrimidin-5-ylidene alditols **337**, representing a 1-oxa-1,3-butadiene system, with dimedone enamines **338** afforded only one enantiomerically pure *cis* diastereoisomer of chromeno[2,3-*d*]pyrimidine-2,4-diones **339** in each reaction (Scheme 53). Analysis of NMR spectra allowed the determination that prepared fused uracils containing amino and enol functional groups exist as a mixture of the neutral form (NF) and zwitterions—dipolar ions (DI). By this simple hetero-Diels–Alder reaction, we can introduce into fused uracil systems such important for biological interaction groups as: different sugar moieties, enol moiety, and different amino groups. The prepared fused uracils contain both amine and enol functional groups, so share amphiprotic properties, and they are zwitterions in solid state [84]. None of the *C*-glycosyl derivatives of chromeno[2,3-*d*]pyrimidines **339** have been examined as inhibitors in the treatment of type 2 diabetes.

**Fig. 15:**
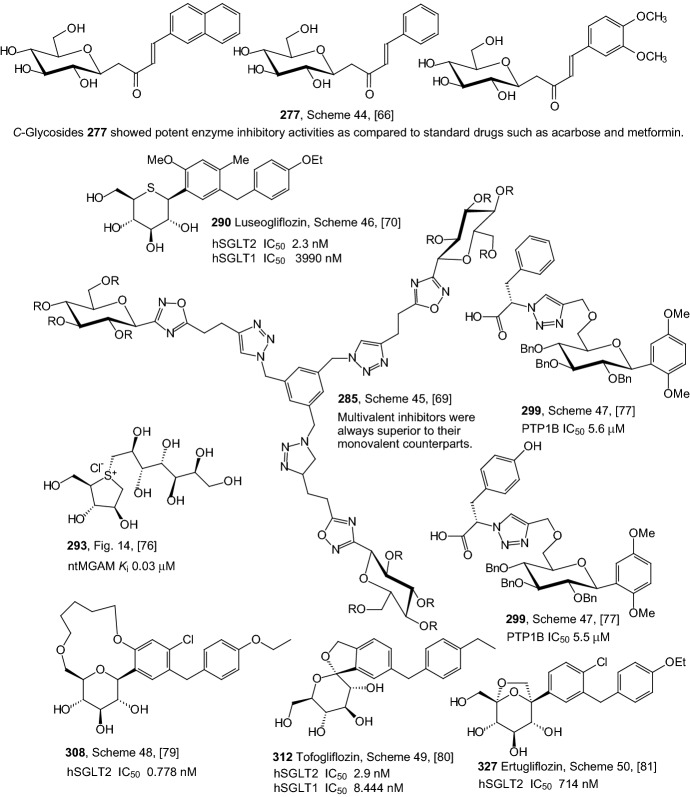
Inhibitory properties of the best inhibitors from the other *C*-glycosyl derivatives described in Sect. 4.3

Figure 15 presents inhibitory properties of the best inhibitors from the other *C*-glycosyl derivatives described in Sect. 4.3. The variety of active inhibitor structures is in this case greater than in the above-mentioned groups of glycosides. For example, aryl butenoyl *C*-glycosides **277** can cause a significant decline in the hyperglycemia of the diabetic rats post sucrose-load. 5-Thioglucose—derived inhibitor **290**, which has a sulfur atom in place of the oxygen atom in the glucose ring, is known as luseogliflozin and it is an orally active SGLT2 inhibitor for the treatment of patients with type 2 diabetes mellitus. The activity of inhibitors may also be influenced by multivalency. On the basis of tests of trivalent inhibitors activity (for example compound **285**), it was found that valency of the molecules influences slightly the inhibition of the enzyme, whereas the presence of a spacer arm between the core and the pharmacophore moieties does not. Multivalent inhibitors were always superior to their monovalent counterparts. In turn, the natural *C*-glycoside kotalanol **293**, which structures comprise a 1,4-anhydro-4-thio-d-arabinitol core and polyhydroxylated acyclic chain, is the most potent ntMGAM inhibitor reported to date (*K*_i_ = 0.03 μM) and highlights the potential of the salacinol class of inhibitors as future drug candidates. Active inhibitors can also be glycoconjugates, such as triazolyl phenylalanine and tyrosine-aryl *C*-glycoside hybrids (compound **299**). Biological assay identified the glycoconjugates **299** that contain the carboxylic acid and benzyl moieties as more active PTP1B inhibitors compared to their ester and debenzylated counterparts. Also, macrocyclic *C*-glycosides may be active inhibitors. For example, [1, 7] dioxacyclopentadecine macrocycle **308** possessing ethoxyphenyl at the distal ring showed the best in vitro inhibitory activity (IC_50_ = 0.778 nM) against human hSGLT2. Compound **312**—tofogliflozin represents a novel class of SGLT2 inhibitors, which have an *O*-spiroketal *C*-arylglucoside scaffold. In turn, ertugliflozin **327** contains a unique dioxa-bicycle in place of the glucose residue of dapagliflozin, and is distinct from other inhibitors, and shows even better SGLT2 inhibitory activity.

## Directions of the Latest Research in 2018–2019

In order to show the latest research directions, papers from 2018 to 2019 are described in a separate Sect. 5. When searching for articles on subject this publication, it was noted that the number of articles describing the synthesis of new inhibitors decreased, and at the same time the number of publications depicting the extraction of active natural *O*-glycosides from plant materials increased. This trend applies in particular to 2018 and 2019. Sect. 5 presents articles on the latest modifications of the *C*-glycosides structure aimed at increasing their effectiveness as inhibitors. Suitable modifications were made to previously tested *C*-glycosides, in the aglycone structure as well as in the sugar molecule structure. Next, the latest proposals concerning the structure of *N*-glycosides as antidiabetic agents were presented and finally the structures of active *O*-glycosides isolated from plants were presented.

In 2018, Kerru et al. published a review article about current antidiabetic agents and their molecular targets [85]. In this review, authors described the use of heterocyclic scaffolds, which have been evaluated for their biological response as inhibitors against their respective antidiabetic molecular targets over the 5-year period from 2012 to 2017. Investigation reveals a diverse target set which includes protein tyrosine phosphatase 1 B (PTP1B), dipeptidly peptidase-4 (DPP-4), free fatty acid receptors 1 (FFAR1), G protein-coupled receptors (GPCR), peroxisome proliferator activated receptor-g (PPARg), sodium glucose co-transporter-2 (SGLT2), α-glucosidase, aldose reductase, glycogen phosphorylase (GP), fructose-1,6-bisphosphatase (FBPase), glucagon receptor (GCGr), and phosphoenolpyruvate carboxykinase (PEPCK). The article presents the structures of various active heterocyclic compounds, of which glycoside derivatives constitute a small group.

**Scheme 54:**
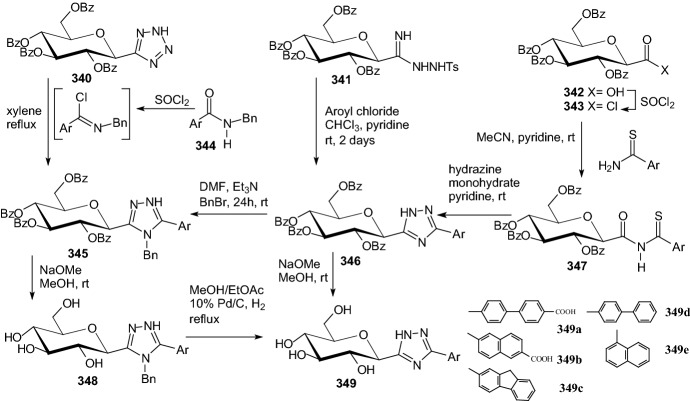
Synthesis of 3-(β-d-glucopyranosyl)-5-substituted-1,2,4-triazoles **349** [86]. The collected yields for individual synthesis steps are not given in the article

Kun and coworkers examined 3-(β-d-glucopyranosyl)-5-substituted-1,2,4-triazoles, which have been revealed as an effective scaffold for the development of potent glycogen phosphorylase inhibitors [86]. The potency of these compounds is very sensitive to the nature of the alkyl/aryl 5-substituent. Authors have chosen for synthesis nine predicted candidates after in silico screening of 2335 new analogues. The compounds **349** were prepared in *O*-perbenzoylated forms by either ring transformation of 5-β-d-glucopyranosyl tetrazole **340** by *N*-benzyl-arenecarboximidoyl chlorides, ring closure of *C*-(β-d-glucopyranosyl)formamidrazone **341** with aroyl chlorides, or that of *N*-(β-d-glucopyranosylcarbonyl)arenethiocarboxamides **347** by hydrazine, followed by deprotections (Scheme 54). Five compounds had *K*i’s < 10 μM (**349 a**–**e**) with potent low μM inhibitors (rmGPa, hlGPa) and three of these (**349a**–**c**) on the submicromolar range for rmGPa [86]. The 3-(β-d-glucopyranosyl)-5-substituted-1,2,4-triazoles described by Kun et al. are predicted to have drug-like potential with only permeability flagged as a potential issue to efficacy.

**Fig. 16:**
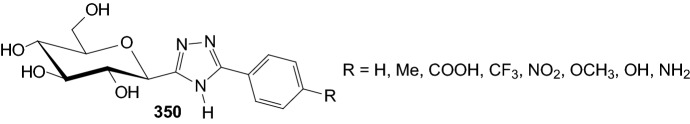
Structure of 3-(β-d-glucopyranosyl)-5-phenyl-1, 2, 4-triazoles **350** [87]

In 2018, Kyriakis et al. studied the inhibitory effect of different groups, in size and hydrophobicity, at the* para* position of 3-(β-d-glucopyranosyl)-5-phenyl-1, 2, 4-triazoles **350** in hlGP by kinetics and X-ray crystallography (Fig. 16) [87]. The most bioactive compound was the one with an amine substituent to show a *K*i value of 0.43 μM. The best C-β-d glucopyranosyl triazole GP inhibitor reported thus far is the compound which has a 2-napthyl group and displays a *K*i value of 0.172 μM for the liver enzyme. Structural studies have revealed the physicochemical diversity of the β-pocket providing information for future rational inhibitor design studies. Comparison of the *K*i values of the inhibitors studied and their structural mode of binding revealed that the addition of the* para* group led to significant increments in potency only when this group exploited hydrophilic or hydrophobic interactions within the β-pocket [87].

**Scheme 55:**
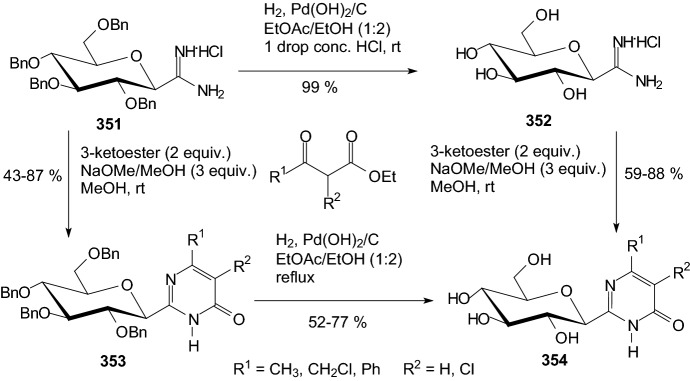
Synthesis of 2-*C*-(β-d-glucopyranosyl)-pyrimidin-4(3*H*)-ones **354** [88]

Szennyes et al. performed a systematic study on the preparation of 2-*C*-(β-d-glucopyranosyl)pyrimidines [88]. Pinner-type cyclization of *O*-perbenzylated *C*-(β-d-glucopyranosyl)formamidine **351** with β-ketoesters, dimethyl malonate, and β-diketone-derived α,β-unsaturated β-chloroketones followed by catalytic hydrogenation resulted in various substituted 2-*C*-(β-d-glucopyranosyl)-pyrimidin-4(3*H*)-ones **354** (Scheme 55), and 2-*C*-(β-d-glucopyranosyl)-4,6-disubstituted-pyrimidines, respectively, in moderate to good yields. These pyrimidine derivatives were also obtained by ring closure of the unprotected *C*-(β-d-glucopyranosyl)formamidine **352** with the same 1,3-dielectrophiles (Scheme 55). A continuous one-pot three-step procedure starting from *O*-peracylated d-glycopyranosyl cyanides was also elaborated to give pyrimidines with various sugar configurations in overall yields (25–94%). These synthetic routes represent the first expansible method to obtain the target compounds. The *C*-glycopyranosyl pyrimidines showed moderate inhibition against α-glucosidase and β-galactosidase enzymes, and no activity against glycogen phosphorylase [88].

**Scheme 56:**
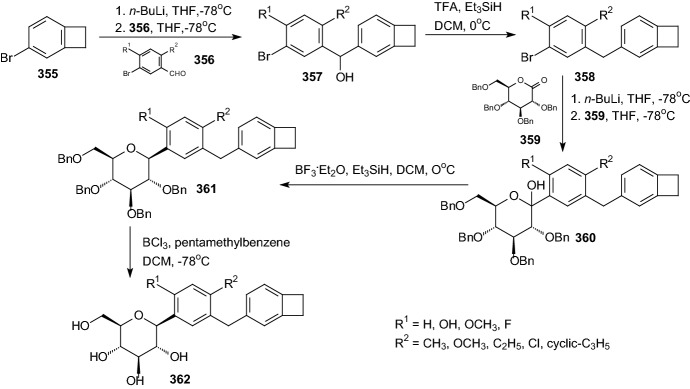
Synthesis of benzocyclobutane-*C*-glycosides **362** [89]. The collected yields for individual synthesis steps are not given in the article

Kuo et al. identified a good-in-class SGLT1/SGLT2 dual inhibitor to improve blood glucose control in type 2 diabetes [89]. The synthesis of benzocyclobutane-*C*-glycosides **362** is described in Scheme 56. Lithium-halogen exchange of compound **355** with *n*-BuLi, followed by the aldol condensation with compound **356** formed **357**. Reduction of compound **357** with TFA/Et_3_SiH gave **358**. Treatment of **358** with *n*-BuLi, followed by the condensation with lactone **359**, resulted in the formation of lactol **360**. Removal of the 1-OH group with BF_3_.Et_2_O/Et_3_SiH provided compound **361**. Deprotection of the benzyl-protecting groups with BCl_3_/penta-methylbenzene gave benzocyclobutane-*C*-glycosides **362**. The biological experiments were carried out on mice, rats, dogs, and monkeys. The best inhibitor **362** (R^1^=H, R^2^=Cl) displayed very high inhibitory potency at both SGLT1 (IC_50_ = 45 nM) and SGLT2 (IC_50_ = 1 nM). New compounds have high in vivo efficacies in different animal model species [89].

**Scheme 57:**
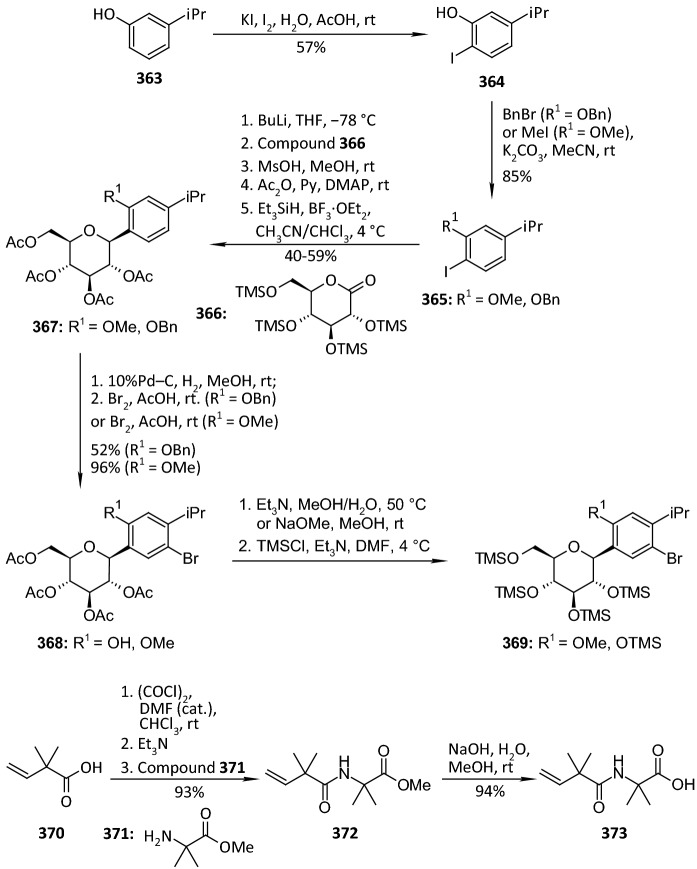
The synthesis of potent, low-absorbable SGLT1 inhibitor [90] (part 1)

**Scheme 58:**
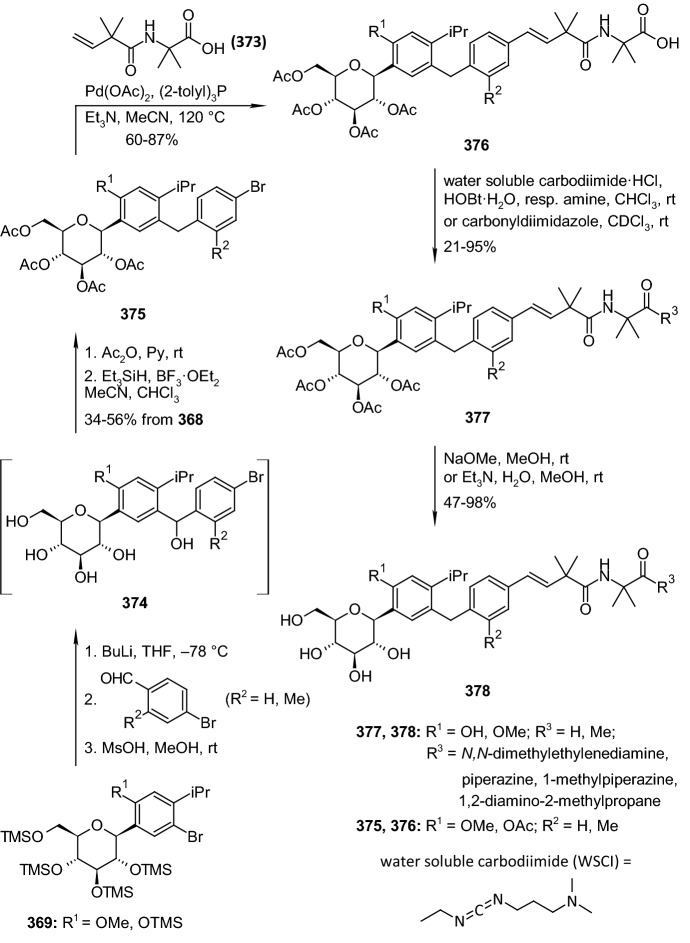
The synthesis of potent, low-absorbable SGLT1 inhibitor **378** [90] (part 2)

In 2019, Kuroda and co-authors reported a discovery of an SGLT1 inhibitor *C*-phenyl d-glucitol derivative **378** (R^1^=OMe, R^2^=H, R^3^=*N,N*-dimethylethylenediamine) (Scheme 57 and 58), with a glucose-lowering effect at a dose of 0.3 mg/kg (p.o.) in Sprague–Dawley (SD) rats [90]. The authors’ aim was to obtain a derivative that excretes mostly in bile to avoid retention of the drug in kidneys. The way of achieving this was imparting greater lipophilicity into the molecule by balancing *C*logP and topological polar surface area (TPSA) together with the absorbability. The inhibitor **378** was obtained in a multi-step synthesis in which as staring materials were used: 3-isopropylphenol **363**, lactone **366**, and the compounds **370** and **371** (Schemes 57 and 58). Compound **378** (R^1^=OMe, R^2^=H, R^3^=*N,N*-dimethylethylenediamine) showed hSGLT1 IC_50_ = 29 nM, hSGLT2 IC_50_ = 20 nM, *C*logP = 3.66, and TPSA = 161 Å^2^. The authors concluded that the compound **378** could potentially be useful as a therapeutic agent for patients with T2DM [90].

**Scheme 59:**
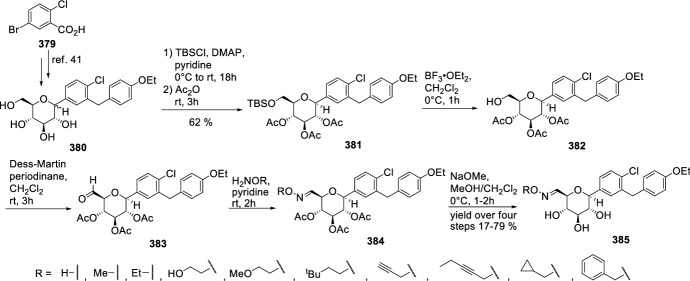
Synthetic pathway of oxime ether derivatives **385** [91]

In the next two articles discussed in the review, attempts were made to synthesize active inhibitors by modifying the sugar part of the *C*-glycoside. In 2018, Yuan and coworkers published an article which concerned the synthesis of 27 aryl *C*-glycosides bearing a C=N/C–N linkage at the glucosyl C6 position [91]. All of these compounds were tested for their inhibitory activity against sodium-dependent glucose co-transporter 2 (SGLT2). Among all obtained oxime ether derivatives **385**, oxime (R=H) showed the best in vitro inhibitory activity (hSGLT2 EC_50_ = 46 nM, hSGLT1 EC_50_ = 3576 nM) and moreover no significant cytotoxicity and low human ether-à-go–go-related gene (hERG) inhibition. The mentioned oxime ether derivatives **385** were prepared starting from 5-bromo-2-chloro-benzoic acid **379** as outlined in Scheme 59. A mixture of α- and β-*C*-glucosides **380** was obtained in accordance with the procedure described in [41]. The next step included regioselective 6-*O*-silylation and per-*O*-acetylation of **380** to synthesize the fully protected β-*C*-glucoside **381**. Then a reaction with boron trifluoride etherate in CH_2_Cl_2_ and oxidation with Dess–Martin periodinane were carried out to introduce the aldehyde at C6 position. At the end, a condensation of aldehyde **383** with different hydroxylamines in pyridine, followed by deacetylation under Zemplén conditions led to obtain the desired oxime ether derivatives **385** [91].

**Scheme 60:**
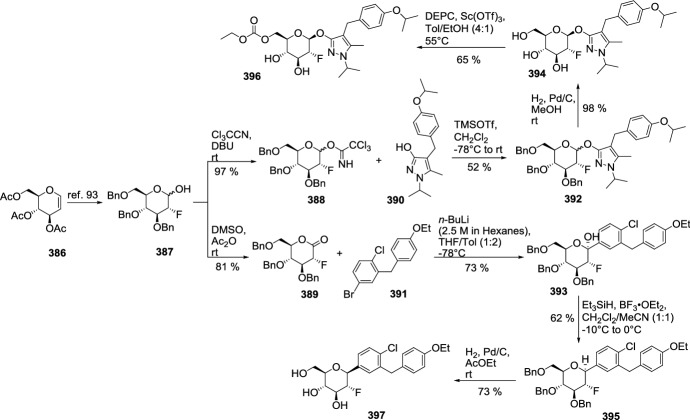
Synthesis of remogliflozin etabonate and dapagliflozin analogues **396** and **397** [92]

The next article from 2018 from Sadurní et al. concerned the influence of molecular editing with fluorine at the C2 position of the pyranose ring of phlorizin analogues to concurrently direct β-selective glycosylation [92]. The authors proposed the methodology of fluorine-directed glycosylation to synthesize the selective SGLT2 inhibitors for type 2 diabetes. The mentioned phlorizin analogues were remogliflozin etabonate and dapagliflozin. These compounds, **396** and **397**, containing fluorine at C2 position were prepared starting from triacetyl-d-glucal **386** as outlined in Scheme 60. A derivative **387** was obtained by the method described in Ref. [93]. The two glycosyl donors **388** and **389**, required to access both target scaffolds, were formed according to the procedures presented in Scheme 60. The next steps in the synthesis of remogliflozin etabonate analogue included: glycosylation, benzyl-deprotection, and selective creation of the primary carbamate. Finally, the remogliflozin etabonate surrogate **396** was obtained. In the case of dapagliflozin surrogate **397**, the next steps involved the activation of **391** by halogen-lithium exchange and addition to the donor lactone **389**, reduction with triethylsilane and BF_3_·OEt_2_, benzyl-deprotection. Based on the conducted biological tests, it was found that the fluorinated dapagliflozin analogue **397** better selectively inhibited human SGLT2 over SGLT1 than remogliflozin etabonate analogue **396** [92].

**Scheme 61:**
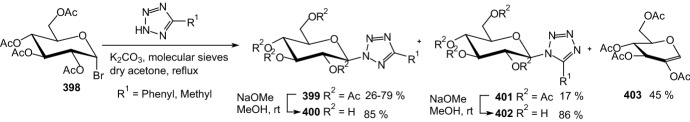
Synthesis of the best inhibitor 2-β-d-glucopyranosyl 5-substituted tetrazole **400** [94]

Kun et al. extended the structure–activity relationships of β-d-glucopyranosyl azole type inhibitors and revealed the extreme sensitivity of such type of inhibitor towards the structure of the azole moiety [94]. Actually, these compounds are the best glucose analogue inhibitors of GP known to date. Their efficiency, among other factors, is due to the formation of an H-bridge between the heterocycle and the His-377 main chain carbonyl group in the active site of the enzyme [94]. 1-Aryl-4-β-d-gluco-pyranosyl-1,2,3-triazoles were prepared by copper-catalyzed azide-alkyne cycloadditions between *O*-perbenzylated or *O*-peracetylated-β-d-glucopyranosyl ethynes and aryl azides. 1-β-d-Gluco-pyranosyl-4-phenyl imidazole was obtained in a glycosylation of 4(5)-phenylimidazole with *O*-peracetylated α-d-glucopyranosyl bromide. *C*-β-d-Glucopyranosyl-*N*-substituted-tetrazoles were synthesized by alkylation/arylation of *O*-perbenzoylated 5-β-d-glucopyranosyl-tetrazole or from a 2,6-anhydroheptose tosylhydrazone and arenediazonium salts. 5-Substituted tetrazoles were glycosylated by *O*-peracetylated α-d-glucopyranosyl bromide **398** to give *N*-β-d-glucopyranosyl-*C*-substituted-tetrazoles **399** and **401** (Scheme 61) [94]. Standard deprotections gave test compounds that were assayed against rabbit muscle glycogen phosphorylase b. Most of the compounds proved inactive; the best inhibitor was 2-β-d-glucopyranosyl-5-phenyltetrazole **400** (R^1^=Ph) (IC_50_ 600 μM).

**Scheme 62:**
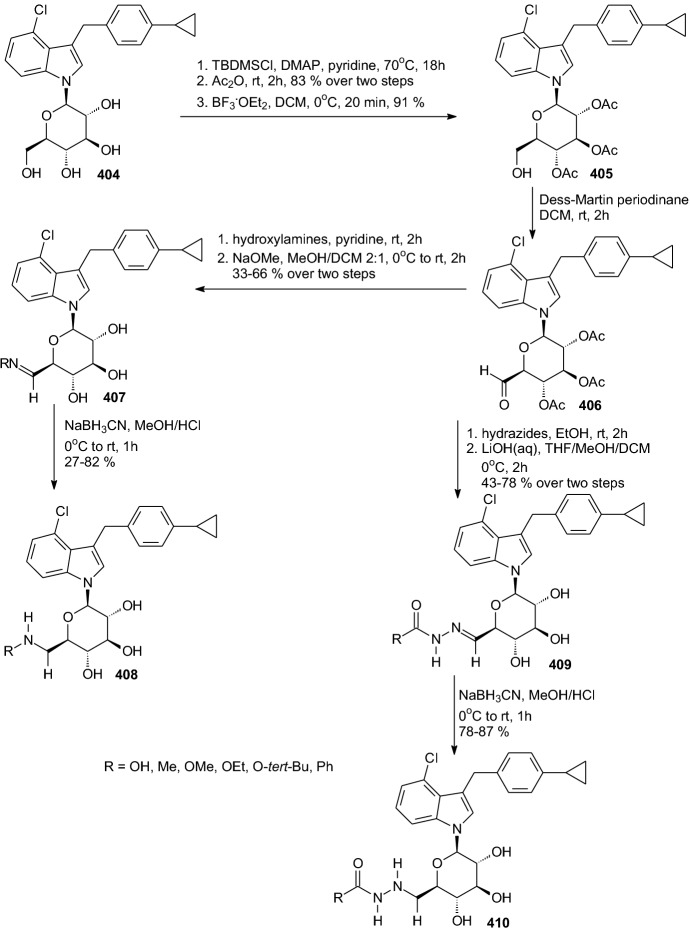
Synthesis of *N*-glucosyl indole derivatives **408** and **410** [95]

Scheme 62 presents *N*-glucosyl indole derivatives, which were designed and synthesized by Chu et al. in 2019 as sodium-dependent glucose co-transporter 2 inhibitors [95]. The aim of the research was to check how modifications in the sugar part of the *N*-glucosyl indoles will affect their inhibitory properties. The synthesis of compounds **408** and **410** started from *N*-glucosyl indole **404**. 6-Aldehyde **406** was obtained by selective protection of the primary alcohol **404** with *tert*-butylchlorodimethylsilane (TBDMSCl), followed by immediate peracetylation by addition of acetic anhydride. Prepared the fully protected *N*-glucoside underwent desilylation under acidic conditions yielded the free primary alcohol **405**. Dess–Martin periodinane (DMP) oxidation gave the desired aldehyde **406**. Condensation of **406** with hydroxylamines and hydrazides followed by deacetylation gave oxime ethers **407** and *N*-acylhydrazones **409**. Reduction products, hydroxylamine *N*-glucosyl indoles **408** and hydrazide *N*-glucosyl indoles **410**, were also synthesized using sodium cyanoborohydride (NaBH_3_CN) under acidic conditions. Authors studied inhibitory activities (EC_50_) of all synthesized *N*-glucosyl indole derivatives **407**, **408**, **409**, **410**, which were determined by measuring the inhibition by uptake of [^14^C]-labeled α-methyl-d-glucopyranoside into hamster ovary cells stably expressing human hSGLT2 or hSGLT1 [95]. The compounds **407** and **409** had similar potency (EC_50_ = 212–286 nM) except **409** (R=OEt, EC_50_ = 1162 nM) and **409** (R=O-*tert*-Bu, EC_50_ = 867 nM). Compound **409** (R = Ph, EC_50_ = 258 nM) was the most potent inhibitor of SGLT2. The next step was the examination of hydroxylamine derivatives **408** and hydrazides **410**. The products **408** and **410** are better inhibitors than substrates **407** and **409**. The hydroxylamine derivatives **408** have similar potency (EC_50_ = 45–294 nM). Taking into account the group of hydrazides **410**, the most activite for hSGLT2 was compoud **410** (R=Me, EC_50_ = 33 nM). Other hydrazides **410** had their power within range (EC_50_ = 63–1761 nM). Compound **410** (R=Me) was advanced into selectivity, pharmacokinetic, and in vivo glucosuria studies. Unfortunately, it was found that this hydrazide has poor pharmacokinetic properties and increases glucosuria in rats only at high doses [95].

A variety of medicinal plants and their active compounds have been used to treat diabetes and related chronic disorders since ancient times. Recently, there is a growing interest in developing natural antidiabetic drugs to manage diabetic complications, especially from plant sources. Thus, due to the return to nature, attention was again paid to the structure of natural *O*-glycosides with antidiabetic action.

**Fig. 17:**
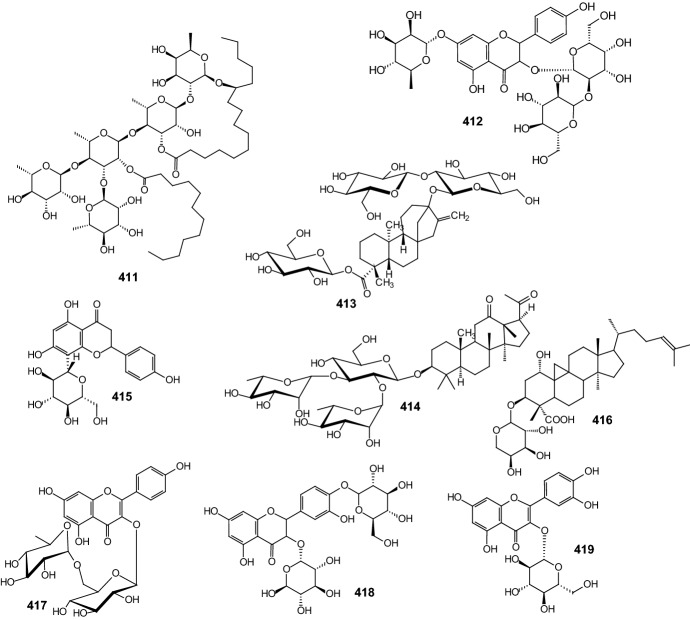
Structures of natural *O*-glycosides with antidiabetic action isolated from plant sources: **411** (morning glory family) [96], **412** (seeds of* Lens culinaris* Medikus) [97], **413** (leaves of* Stevia rebaudiana* bertoni) [98], **414** (*Gynostemma longipes*) [99], **415** (*Ficus* species) [100], **416** (*Leea indica*) [101], **417** (Lu’an GuaPian tea-* Camellia sinensis* L.O. Kuntze) [102], **418** (onion solid waste) [103], **419** [104]

In 2018, Rosas-Ramírez et al. postulated that resin glycosides from the morning glory family (Convolvulaceae) may be a source of phytotherapeutic agents with antihyperglycemic properties for the prophylaxis and treatment of non-insulin-dependent type 2 diabetes mellitus [96]. Twenty-seven individual resin glycosides were evaluated for their α-glucosidase inhibitory potential. Four of these compounds displayed an inhibitory activity comparable to acarbose. In Fig. 17, compound **411** presents the structure of one of the four active glycosides isolated from the morning glory family. Based on molecular modeling studies performed by docking analysis, it was predicted that the active compounds and acarbose bind to the α-1,4-glucosidase enzyme catalytic site of MAL12 from the yeast* Saccharomyces cerevisiae* through stable hydrogen bonds primarily with the amino acid residues HIS279 and GLN322 [96]. Docking studies with the human maltase-glucoamylase (MGAM) also identified binding modes for resin glycosides inside the catalytic site in the proximity of TYR1251. Resin glycosides could help to control postprandial glucose levels due to their inhibitory activity of α-glucosidases, which play a crucial role in the production of glucose [96].

In 2018, Kim and coworkers isolated from the seeds of* Lens culinaris* Medikus (Fabaceae) three flavonol glycosides and tested them for their DPP-IV–inhibitory activity [97]. Figure 17 contains the structure of one of the three active glycosides (compound **412**) isolated from the morning glory family. Dipeptidyl peptidase IV (DPP-IV) is a new target for the treatment of type 2 diabetes mellitus. DPP-IV inhibitors shorten the inactivation of glucagon-like peptide 1 (GLP-1), permitting the incretin to stimulate insulin release, thereby combating hyperglycemia. It was demonstrated for the first time that three isolated flavonol glycosides inhibited DPP-IV activity in a concentration-dependent manner in vitro bioassay system [97]. Molecular docking experiments of these compounds within the binding pocket of DPP-IV were also conducted. All investigated compounds readily fit within the active sites of DPP-IV, in low-energy conformations characterized by the flavone core structure having optimal electrostatic attractive interactions with the catalytic triad residues of DPP-IV.

The use of steviol glycosides as non-caloric sweeteners has proven to be beneficial for patients with type 2 diabetes mellitus, obesity, and metabolic syndrome [98]. Figure 17 shows the structure of one of the natural antidiabetic *O*-glycosides **413** isolated from* Stevia* leaves. Recent data also demonstrate that steviol and stevioside might act as glucocorticoid receptor (GR) agonists and thus correlate with adverse effects on metabolism. In 2018, Panagiotou et al. provided strong evidence that steviol and steviol glycosides exert GR-mediated effects in cancer Jurkat cells [98].

In 2018, Pham and coworkers examined the chemical composition of* Gynostemma longipes*, an ethnomedicinal plant used to treat type 2 diabetes mellitus by local communities in Vietnam [99]. Ten new dammarane triterpenes, including two hexanordammarane glycosides and five other dammarane glycosides, were isolated from a ethanolic extract of the whole *G. longipes* plant. The structures of the new compounds were elucidated using diverse spectroscopic methods. All of the isolates were evaluated for their stimulatory activities on glucose uptake. Compound **414** (Fig. 17) showed particularly potent stimulatory effects [99].

Deepa et al. presented in the review paper that extracts from various species of* Ficus* and isolated compounds significantly have enhanced insulin secretion and subsequently reduced blood glucose level [100]. *O*-Glycosides (for example compound **415**, Fig. 17) isolated from* Ficus* species exhibited remarkable antidiabetic properties.

In 2018, Kekuda et al. prepared a comprehensive review about traditional uses, chemistry and pharmacological activities of *Leea indica* (Burm. f.) Merr.(Vitaceae) [101]. The plant *L. indica* is traditionally used, inter alia, as a medicine for diabetes. Hypoglycemic activity of alcoholic and hydroalcoholic extracts of *L. indica* leaves using glucose tolerance test and alloxan-induced diabetes model in rats, was evaluated. The extract administration significantly reduced blood glucose levels, indicating hypoglycemic activity of leaf extracts [101]. Figure 17 shows the structure of one of the natural *O*-glycosides (mollic acid arabinoside **416**) that have been isolated from the plant *L. indica*.

In another paper from 2018, Hua and coworkers have proven that green tea may favorably modulate blood glucose homeostasis, and regular consumption of green tea can prevent the development of type 2 diabetes mellitus [102]. Authors described the inhibition of α-glucosidase and α-amylase by flavonoid glycosides from Lu’an GuaPian tea. The kaempferol monoglycoside showed inhibitory activity against α-glucosidase with IC_50_ at 40.02 ± 4.61 μM, and kaempferol diglycoside (Fig. 17, *O*-glycoside **417**) showed α-amylase inhibition with IC_50_ at 0.09 ± 0.02 μM [95]. Application of Lu’an GuaPian green tea as a functional food ingredient can regulate postprandial hyperglycemia through inhibition of α-glucosidase/α-amylase by the mono and diglycosides of kaempferol [102].

Nile et al. described valorization of onion solid waste and their flavonols for assessment of cytotoxicity, enzyme inhibitory, and antioxidant activities [103]. Onion (*Allium cepa* L.) is rich in flavonols like quercetin and quercetin glycosides. These glycosides have been extracted and tested against enzymes of clinical importance in diabetes. The samples exhibited significant antidiabetic effects. Results indicated that OSW (onion solid waste) and flavonol glycosides are potential antidiabetic agents [103]. Figure 17 shows the structure of one of the natural *O*-glycosides (quercetin-3,4′-*O*-diglucoside QDG **418**) that have been isolated from onion solid waste.

Jayachandran and coworkers have designed studies to accumulate the experimental evidence in support of antidiabetic effects of isoquercetin **419** (Fig. 17) [104]. Supplementation with isoquercetin significantly normalized blood sugar levels, insulin, and regulated the mRNA expression of insulin signaling genes and carbohydrate-metabolizing enzyme genes. The results achieved with isoquercetin are similar to that of the standard drug glibenclamide [104]. The findings suggest isoquercetin could be a possible therapeutic agent for treating diabetes mellitus in the near future.

## Biological Action of the Glycosides Described in this Review and Patents of Marketed Drugs

At present, the normalization of glycemia in diabetes is a rather complicated and problematic issue of diabetology. Despite the growing knowledge of various chemical and biochemical aspects of diabetes mellitus, the specific molecular mechanisms leading to T2DM are still unknown. Current pharmacological treatments are symptomatic and aim at maintaining blood glucose levels close to the fasting normoglycemic range of 3.5–6 mM/l. A large number of oral antidiabetic drugs, which exert their effects through various mechanisms, aimed at eliminating three major metabolic disorders leading to hyperglycemia: dysfunction of β-cells, peripheral insulin resistance, excessive hepatic glucose production [7]. Promising direction for design of new drugs for the treatment of diabetes mellitus is the regulation of key carbohydrate metabolism enzymes: inhibition of glycogen phosphorylase (GP), sodium-dependent glucose transporters (SGLT), and protein tyrosine phosphatase (PTP).

Depending on the binding site of the enzyme molecule, GP inhibitors are divided into compounds that block the catalytic site and compounds that block the allosteric sites [105, 106]. The following groups of chemical compounds were studied as inhibitors of the catalytic site of GP: *N*-acyl-β-(d-glucopyranosylamine derivatives, *N*-β-d-glucopyranosyl ureas, 2-(d-glucopyranosyl)-5-methyl-1,3,4-oxadiazole derivatives, 2-(d-glucopyranosyl)-benzimidazoles, 3-substituted 5-β-d-glucopyranosyl-1,2,4-oxadiazoles, glucopyranosyliden-spiro-thiohydantoins. Efforts in identifying the best heterocyclic junction between the glucose and pharmacophore units were patented, taking into account 1,2,4-triazole and imidazole moieties [107].

**Fig. 18:**
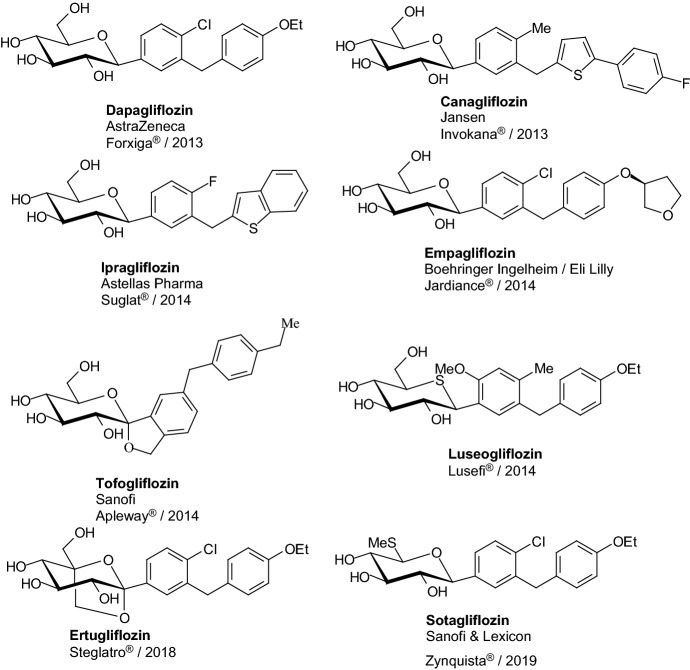
SGLT2 inhibitors approved as marketed drugs. Name, company, brand name, and year for approval are provided

Inhibitors of sodium-dependent glucose co-transporter 2 (SGLT2) are an attractive method of type 2 diabetes treatment because of their distinct mechanism of action, in which blood glucose levels are reduced independently of insulin secretion [108]. In healthy individuals, greater than 99% of the plasma glucose that is filtered in the kidney is reabsorbed, resulting in less than 1% of the total filtered glucose being excreted in urine [41]. This reabsorption process is mediated by two sodium-dependent glucose cotransporters (SGLTs): SGLT1, a low-capacity, high-affinity transporter and SGLT2, a high-capacity, low-affinity transporter that is expressed mainly in the kidney [41]. It is estimated that 90% of renal glucose reabsorption is facilitated by SGLT2 residing on the surface of the epithelial cells lining the S1 segment of the proximal tubule; the remaining 10% is likely mediated by SGLT1 localized on the more distal S3 segment of the proximal tubule [41]. Several selective SGLT2 inhibitors have been developed by structural modification of phlorizin, the first known SGLT inhibitor. *C*-Linked β-glycosides (gliflozins) are at advanced stages of development because of their metabolic stability, high oral bioavailability, and plasma exposure. Of these, dapagliflozin, canagliflozin, ipragliflozin, empagliflozin, tofogliflozin, luseogliflozin, ertugliflozin, and sotagliflozin have been approved for the treatment of type 2 diabetes mellitus recently [108]. These compounds are now approved as marketed drugs (Fig. 18). For example, inhibition of SGLT2 with Forxiga (dapagliflozin) reduces renal glucose reabsorption and thereby increases urinary glucose exretion [109]. From 2014, Forxiga is indicated by the FDA as an adjunct to diet and exercise to improve glycemic control in adults with type 2 diabetes and is not recommended for patients with type 1 diabetes mellitus.

Of particular importance to the downregulation of insulin signaling is protein tyrosine phosphatase 1B (PTP1B), which dephosphorylates the receptor (IR) on the surface of a cell [110]. Inhibition of PTP1B may represent a practical strategy for the treatment of type 2 diabetes [111]. Various strategies have been developed to design and synthesize potent and selective PTP1B inhibitors. The principal approach is based on mimicking the phosphotyrosine moiety. Many PTP inhibitors contain a quinone functionality. Series of *C*-glucosyl 1,4-benzo- and 1,4-naphtho-quinones were found as better PTP1B inhibitors than their analogues displaying 1,4-dimethoxybenzene or -naphthalene residues [40]. Modifications of molecules at the primary 6-position were made by transformation to either carboxylic, azido, or benzamido groups, or elongation by azide-alkyne click chemistry to triazole ring formation [112].

## Conclusions

In this review, we disclosed studies on the preparation of glycosides that can be applied as inhibitors of glycogen phosphorylase, sodium glucose cotransporter 2, protein tyrosine phosphatase 1B and other more specific enzymes. The natural product phlorizin was identified as the first type 2 diabetes mellitus inhibitor. Because *O*-glycosides are usually hydrolytically unstable, many carbohydrate analogues such as *N*-glycosides and *C*-glycosides have been synthesized and used as enzyme inhibitors. In comparison to *O*-glycosides, the *C*-glycosides are structurally more stable against acidic and enzymatic cleavage due to the existence of their C–C glycosidic bond. Among glycomimetics, *C*-glycosyl compounds have attracted much attention. By use of structure–activity relationships, several new glycosides have been developed as inhibitors and studied in clinical trials. Some of the described glycoside-based molecules, for example dapagliflozin, canagliflozin, ipragliflozin, empagliflozin, tofogliflozin, luseogliflozin, ertugliflozin, and sotagliflozin, have been approved as marketed drugs.

**Fig. 19:**
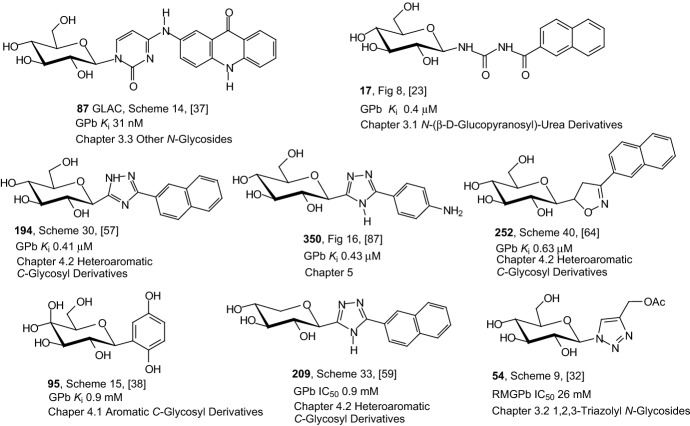
The best GPb inhibitors selected from the figures summarizing the activity of inhibitors discussed in a given Sects

Figure 19 shows the best GPb inhibitors described in this article, which were selected from Figs. 7, 9, 10, 11, 13, and 15 presenting activity of inhibitors having a specific *O*, *N*, or *C*-glycoside structure and from Sect. 5 describing directions of the latest research from 2018 to 2019. Below each compound there is information about what type of glycoside it represents. The compounds were ranked in order from the inhibitor with the highest activity (the lowest *K*_i_) to the inhibitor with the lowest activity (the highest *K*_i_). Where the *K*_i_ values were not reported in the publication, the inhibitory properties were compared based on the IC_50_ values. Seven of the eight inhibitors shown in Fig. 19 are derivatives of d-glucose. Changes in the sugar configuration as well as removal or replacement of substituents of the glucose moiety proved detrimental for the inhibition. The crucial role of the aglycon in the efficiency of the inhibitors is also presented in this article. Based on the analysis of the structure of compounds that are the best GPb inhibitors, it can be concluded that a high activity is ensured by the presence of the 2-naphthol group. Four of the eight compounds shown in Fig. 19 have a 2-naphthol group. Analysis of the structures of the best GPb inhibitors (Fig. 19) leads to the conclusion that the highest activity is possessed by inhibitors containing, as aglycones, heterocyclic rings with nitrogen atoms.

**Fig. 20:**
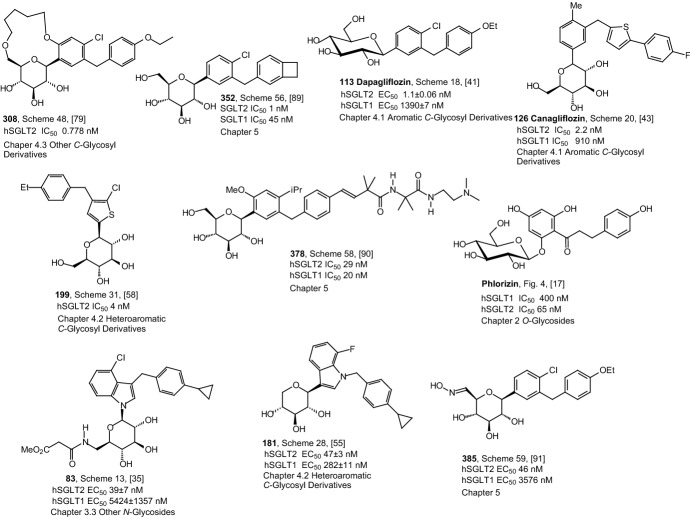
The best SGLT2 inhibitors selected from the figures summarizing the activity of inhibitors discussed in a given Sects

In turn, Fig. 20 presents the best SGLT2 inhibitors. They were selected from Figs. 7, 9, 10, 11, 13, and 15 as representatives of glycosides with the structure described in the given Sects and from Sect. 5 describing directions of the latest research from 2018 to 2019. The compounds were arranged in order from the best inhibitor (the lowest IC_50_) to the inhibitor with lower activity (the highest IC_50_). The activity of inhibitors for which no IC_50_ values were reported in the publication was evaluated by comparing the EC_50_ values. Analysis of the structure of the best SGLT2 inhibitors allows the conclusion that not only glucose but also its derivatives or xylose provide inhibitory activity. Looking at the structure of active SGLT2 inhibitors from Fig. 20, it can also be seen that each of them has one or two phenyl groups in its structure. Considering the structure of the aglycone, one cannot distinguish a specific heteroaromatic system whose structure would be repeated in the structure of the inhibitors considered. However, it can be seen that in two cases there is a thiophene ring and in the other two an indole system. Eight of the ten SGLT2 inhibitors shown in Fig. 20 have a chlorine or fluorine atom in their structure.

The conclusions drawn from the analysis of the structure of the best inhibitors should be taken into account when designing antidiabetic drugs. We sincerely hope that this article will stimulate further research in glycoside derivatives synthesis and will encourage scientists to design novel inhibitors in the treatment of type 2 diabetes mellitus.

## Abbreviations

Ac: Acetyl
Ar: Aryl
Asn: Asparagine
Asp: Aspartic acid
Bn: Benzyl
Boc: *tert*-Butyloxycarbonyl
*i*Bu: Isobutyl
*n*-Bu: *n*-Butyl
*t*-Bu: *tert*-Butyl
Bz: Benzoyl
CAN: Cerium ammonium nitrate
CuAAC: Copper-catalyzed azide–alkyne cycloaddition
DBU: 1,8-Diazabicyclo[5.4.0]undec-7-ene
DCC: *N,N’*-Dicyclohexylcarbodiimide
DCM: Dichloromethane
DIAD: Diisopropyl azodicarboxylate
DMAP: *N*,*N*-Dimethyl-4-aminopyridine
DME: Dimethoxyethane
DMF: Dimethylformamide
DMSO: Dimethyl sulfoxide
EDCI: *N*-Ethyl-*N*′-(3-dimethylaminopropyl)carbodiimide
Et: Ethyl
GP: Glycogen phosphorylase
GS: Glycogen synthase
Hetaryl: Heteroaromatic ring
HMPA: Hexamethylphosphoramide
HOBt: Hydroxybenzotriazole
HPLC: High-performance liquid chromatography
IC_50_: Half maximal inhibitory concentration
K_i_: Inhibition constant
LDA: Lithium diisopropylamide
Me: Methyl
MOM: Methoxymethyl
MOMCl: Methoxymethyl chloride
Ms: Mesyl
NBS: *N*-bromosuccinimide
NCS: *N*-chlorosuccinimide
NMM: *N*-Methylmorpholine
NMO: *N*-Methylmorpholine *N*-oxide
Ph: Phenyl
iPr: Isopropyl
PTP1B: Protein tyrosine phosphatase 1B
QSAR: Quantitative structure–activity relationship
RMGP: Rat muscle glycogen phosphorylase
SAR: Structure–activity relationship
SGLT: Sodium-dependent glucose cotransporter
TBA: *tert*-Butyl alcohol
TBAF: Tetrabutylammonium fluoride
TBDMS: *tert*-Butyldimethylsilyl
TBDMSCl: *tert*-Butyldimethylsilyl chloride
TBS: *tert*-Butyldimethylsilyl
TBSCl: *tert*-Butyldimethylsilyl chloride
T2DM: Type 2 diabetes mellitus
TEA: Triethylamine
TFA: Trifluoroacetic acid
THF: Tetrahydrofuran
TIPS: Triisopropylsilyl
TMS: Trimethylsilyl
TMSI: Trimethylsilyl iodide
TMSOTf: Trimethylsilyl trifluoromethanesulfonate
Ts: Tosyl
Tyr: Tyrosine
